# Identification of RIP1 as a critical mediator of Smac mimetic-mediated sensitization of glioblastoma cells for Drozitumab-induced apoptosis

**DOI:** 10.1038/cddis.2014.592

**Published:** 2015-04-16

**Authors:** S Cristofanon, B A Abhari, M Krueger, A Tchoghandjian, S Momma, C Calaminus, D Vucic, B J Pichler, S Fulda

**Affiliations:** 1 Institute for Experimental Cancer Research in Pediatrics, Goethe-University, Frankfurt, Germany; 2 Werner Siemens Imaging Center, Department of Preclinical Imaging and Radiopharmacy, Eberhard Karls University, Tuebingen, Germany; 3 Institute of Neuropathology, Goethe-University, Frankfurt, Germany; 4 Genentech, Inc, South San Francisco, CA, USA; 5 German Cancer Consortium (DKTK), Heidelberg, Germany; 6 German Cancer Research Center (DKFZ), Heidelberg, Germany

## Abstract

This study aims at evaluating the combination of the tumor-necrosis-factor-related apoptosis-inducing ligand (TRAIL)-receptor 2 (TRAIL-R2)-specific antibody Drozitumab and the Smac mimetic BV6 in preclinical glioblastoma models. To this end, the effect of BV6 and/or Drozitumab on apoptosis induction and signaling pathways was analyzed in glioblastoma cell lines, primary glioblastoma cultures and glioblastoma stem-like cells. Here, we report that BV6 and Drozitumab synergistically induce apoptosis and reduce colony formation in several glioblastoma cell lines (combination index<0.1). Also, BV6 profoundly enhances Drozitumab-induced apoptosis in primary glioblastoma cultures and glioblastoma stem-like cells. Importantly, BV6 cooperates with Drozitumab to suppress tumor growth in two glioblastoma *in vivo* models including an orthotopic, intracranial mouse model, underlining the clinical relevance of these findings. Mechanistic studies reveal that BV6 and Drozitumab act in concert to trigger the formation of a cytosolic receptor-interacting protein (RIP) 1/Fas-associated via death domain (FADD)/caspase-8-containing complex and subsequent activation of caspase-8 and -3. BV6- and Drozitumab-induced apoptosis is blocked by the caspase inhibitor zVAD.fmk, pointing to caspase-dependent apoptosis. RNA interference-mediated silencing of RIP1 almost completely abolishes the BV6-conferred sensitization to Drozitumab-induced apoptosis, indicating that the synergism critically depends on RIP1 expression. In contrast, both necrostatin-1, a RIP1 kinase inhibitor, and Enbrel, a TNF*α*-blocking antibody, do not interfere with BV6/Drozitumab-induced apoptosis, demonstrating that apoptosis occurs independently of RIP1 kinase activity or an autocrine TNF*α* loop. In conclusion, the rational combination of BV6 and Drozitumab presents a promising approach to trigger apoptosis in glioblastoma, which warrants further investigation.

Glioblastoma is the most common primary malignant brain tumor in adulthood.^1^ Treatment response and prognosis are still very poor in this malignancy despite aggressive therapies,^2^ highlighting the urgent need to come up with innovative therapeutic concepts.

Resistance to apoptosis is a characteristic trait of human cancers that contributes to tumorigenesis as well as to treatment resistance.^3^ Apoptosis (programmed cell death) represents the cell's intrinsic suicide program that comprises two key signaling pathways.^4^ The extrinsic (death receptor) pathway is engaged by the crosslinking of death receptors of the tumor necrosis factor (TNF) receptor family such as TRAIL receptors on the cell surface by their corresponding ligands, for example, TRAIL, also known as Apo2L.^5, 6^ This initiates the recruitment of FADD and caspase-8 to the death-inducing signaling complex (DISC) that drives caspase-8 activation.^5^ In the intrinsic (mitochondrial) pathway, mitochondrial intermembrane proteins such as cytochrome c and second mitochondria-derived activator of caspases (Smac) are released into the cytosol, promoting activation of effector caspase-3 via the apoptosome (cytochrome c) or by antagonizing inhibitor of apoptosis (IAP) proteins (Smac).^7^

IAP proteins substantially contribute to apoptosis resistance of human cancers, because they are expressed at high levels in many tumors.^8^ Therefore, IAP proteins are considered as promising anticancer drugs targets. To interfere with aberrant expression and function of IAP proteins, small-molecule antagonists such as Smac mimetics have been designed to mimic the N-terminal part of Smac.^8^ Smac mimetics promote caspase activation by neutralizing the XIAP-imposed inhibition of caspase-3, -7 and -9.^8^ In addition, Smac mimetics stimulate proteasomal degradation of IAP proteins that contain a RING motif with E3 ligase activity such as cellular inhibitor of apoptosis (cIAP) proteins.^9, 10, 11^ Depletion of cIAPs results in reduced ubiquitination of receptor-activating protein 1 (RIP1), which favors the assembly of a RIP1/FADD/caspase-8 complex, leading to caspase-8 activation.^9, 12, 13^ Loss of cIAPs also results in activation of the non-canonical NF-*κ*B pathway via stabilization of NIK and transcriptional activation of TNF*α* as a prototype NF-*κ*B target gene.^9, 10, 14^ Via an autocrine/paracrine mechanism TNF*α* can mediate Smac mimetic-induced apoptosis in cells that have lost cIAP proteins in response to Smac mimetic treatment.^9, 10^ BV6 represents a bivalent Smac mimetic that consists of two Smac mimetics connected by a chemical linker.^9^

Previously, we demonstrated in a proof-of-concept study that Smac peptides can potentiate TRAIL-induced apoptosis in glioblastoma cells.^15^ Compared with this initial study, more advanced, non-peptidic small-molecule IAP antagonists are currently under evaluation in early clinical trials^8^ as well as fully human monoclonal TRAIL receptor antibodies.^16^ As there is increasing evidence showing that monotherapy with either IAP antagonists or TRAIL receptor agonists will likely not be sufficient for optimal antitumor activity in the majority of cancers,^5, 8^ rational combination strategies will become particularly important to exploit the therapeutic potential of these compounds. Therefore, the aim of the present study is to evaluate a rational combination of two novel anti-cancer agents in preclinical models of glioblastoma, that is, the TRAIL-receptor 2 (TRAIL-R2)-specific antibody Drozitumab to directly trigger apoptosis and the Smac mimetic BV6 to lower the threshold for apoptosis induction by antagonizing IAP proteins.

## Results

### BV6 sensitizes glioblastoma cells to Drozitumab-induced cytotoxicity

**Figure 1:**
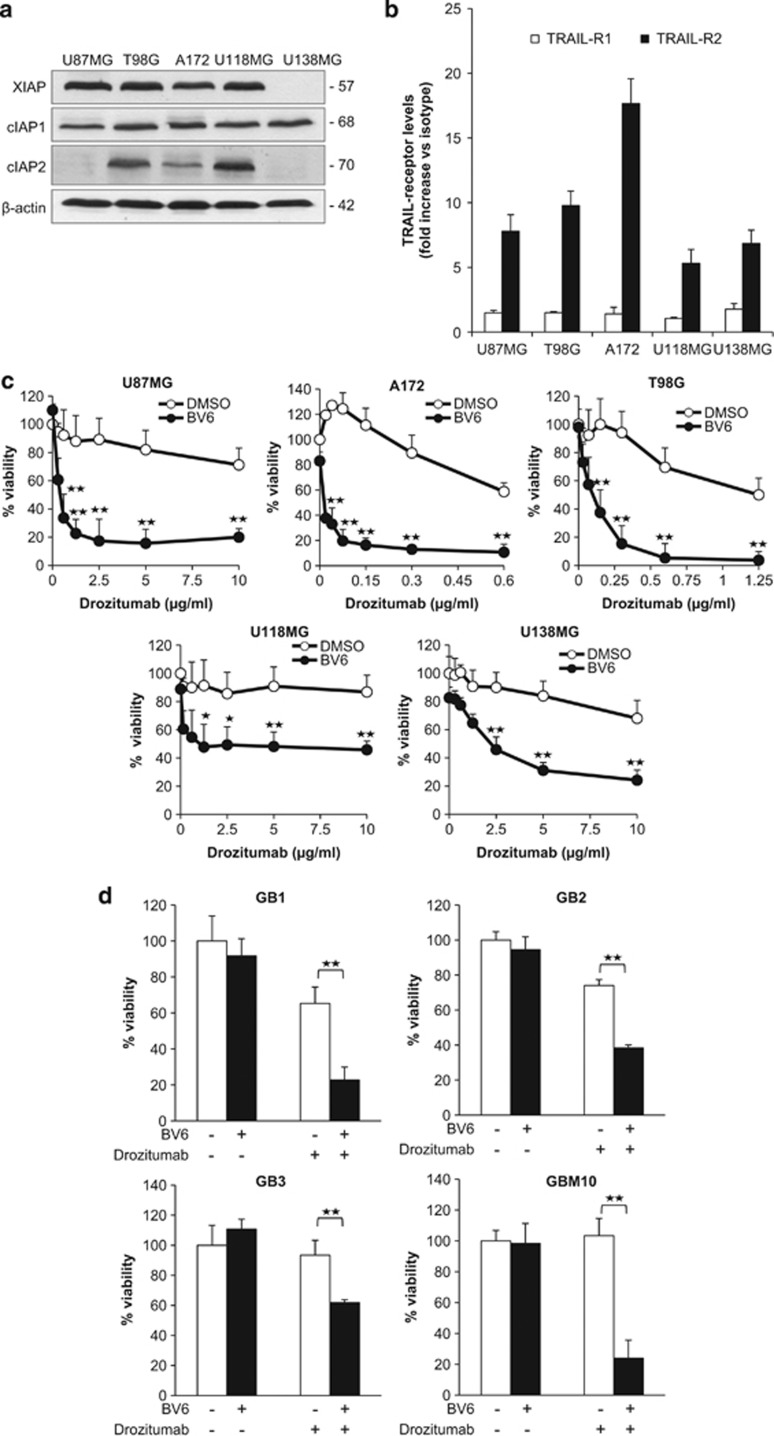

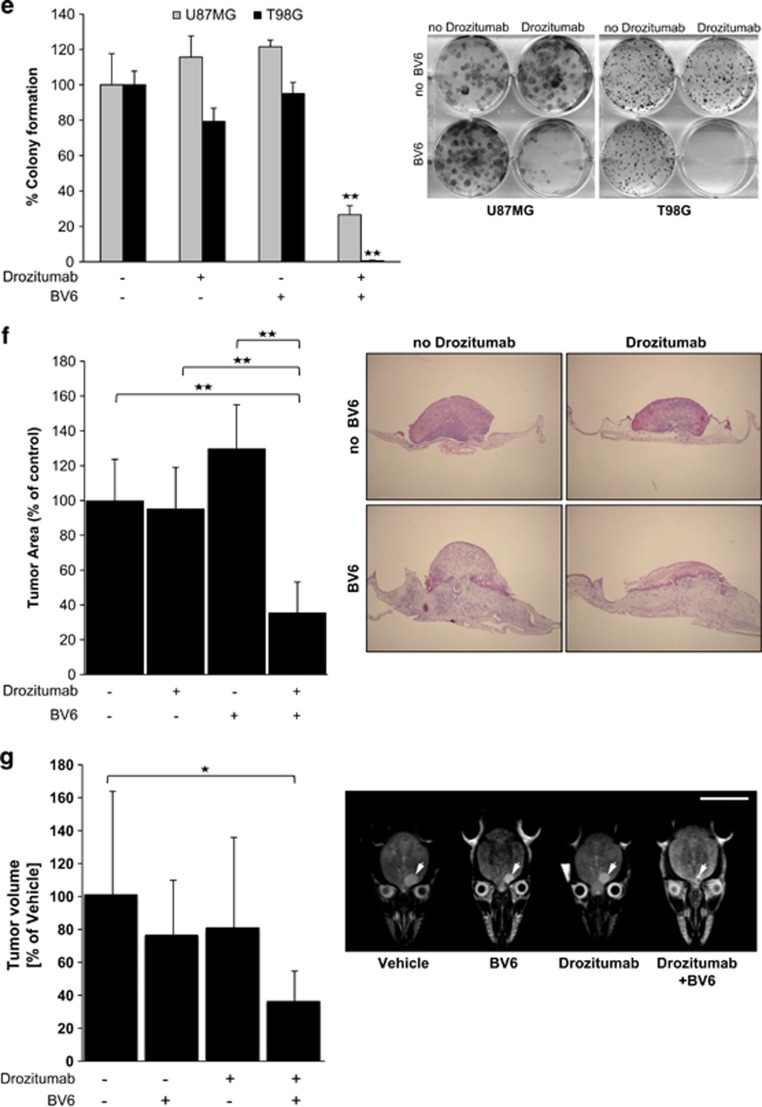
BV6 sensitizes glioblastoma cells to Drozitumab-induced apoptosis. (**a**) Expression levels of cIAP1, cIAP2 and XIAP were assessed by western blot analysis in glioblastoma cell lines. *β*-actin was used as loading control. (**b**) Surface expression of TRAIL-R1 and -R2 on glioblastoma cell lines was determined by flow cytometry. Relative expression levels of TRAIL receptors are shown as fold increase of isotype control. (**c**) Cells were treated for 72 h with indicated concentrations of Drozitumab and/or BV6 (U87MG: 4 *μ*M; A172, U138MG: 3 *μ*M; T98G: 2 *μ*M; U118MG: 1 *μ*M). Cell viability was determined by MTT assay and is expressed as the percentage of untreated controls; mean + S.E.M. values of three independent experiments performed in triplicate are shown; *P<0.05; **P<0.001. (**d**) Primary glioblastoma cells (GB1-3) and glioblastoma stem-like cells (GBM10) were treated for 72 h with BV6 and/or Drozitumab (GB1: 8 *μ*M BV6/0.6 *μ*g/ml Drozitumab, GB2: 2 *μ*M BV6/0.6 *μ*g/ml Drozitumab, GB3: 2 *μ*M BV6/2.5 *μ*g/ml Drozitumab, GBM10: 2.5 *μ*M BV6/10 *μ*g/ml Drozitumab). Cell viability was determined by MTT assay and is expressed as the percentage of untreated controls; mean + S.E.M. values of three independent experiments performed in triplicate are shown; **P<0.001. (**e**) U87MG and T98G cells were treated for 72 h with 5 *μ*g/ml (U87MG) or 0.3 *μ*g/ml (T98G) Drozitumab and/or 4 *μ*M (U87MG) or 2 *μ*M (T98G) BV6 and then seeded as single cells. Colonies were stained with crystal violet after 14 days and were counted under the microscope. One representative experiment of three independent experiments (right panel) and the percentage of colony numbers compared with untreated control are shown (left panel; data represent mean + S.E.M. of three independent experiments (**P<0.001) comparing samples treated with the combination *versus* control samples). (**f**) U87MG cells were seeded on the CAM of chicken embryos and treated with 1 *μ*M BV6 and/or 2.5 *μ*g/ml Drozitumab for 2 days. Tumor growth was analyzed using hematoxylin and eosin-stained paraffin sections of the CAM as described in Materials and Methods. Tumor area as percentage of the untreated control group (left panel; mean + S.E.M. of 18 samples per group of three independent experiments; **P<0.001) and representative pictures of hematoxylin and eosin-stained sections of the CAM (right panel; × 4 magnification) are shown. (**g**) U87MG cells were stereotactically implanted into the brain of nude mice. At days 6 and 9 post implantation of tumor cells 10 *μ*g Drozitumab, 16 nmol BV6, a combination of both compounds or PBS as vehicle were stereotactically injected into tumors. On day 13 post implantation of tumor cells, tumor growth was assessed by MR imaging using a 1 T Icon-Scanner equipped with a mouse brain coil and a T2-weighted image of the brain was acquired. Tumor volumes were determined as described in Materials and Methods. Exemplary MR images are shown in the right panel (scale bar: 1cm) and tumor volumes in the left panel. Mean tumor volume of all animals as percentage of the vehicle group was determined as described in Materials and Methods. At least *n*=5 animals per group were considered. Mean + S.D. are shown, *P<0.05

To investigate whether targeting IAP proteins can prime glioblastoma cells towards TRAIL, we selected a panel of glioblastoma cell lines, which are heterogeneous for *p53* and *PTEN* status (Supplementary Table 1), two key signaling components that are often altered in glioblastoma.^17^ cIAP1 and XIAP were expressed in all tested glioblastoma cell lines except U138MG, whereas the expression of cIAP2 was detected in three out of five cell lines (Figure 1a). Analysis of TRAIL receptor surface expression revealed that TRAIL-R2 was expressed in all studied cell lines in contrast to little expression of TRAIL-R1 (Figure 1b,Supplementary Figure 1A). The latter finding is consistent with the reported frequent epigenetic inactivation of TRAIL-R1 in glioblastoma.^18^

As glioblastoma cell lines predominately express TRAIL-R2, we selected a human monoclonal antibody specifically directed against TRAIL-R2 (Drozitumab) to target the TRAIL signaling pathway.^19^ To antagonize IAP proteins, we used the bivalent Smac mimetic BV6.^9^ Importantly, the addition of BV6 profoundly sensitized glioblastoma cells to Drozitumab-induced loss of cell viability, whereas treatment with Drozitumab alone had a moderate or minor effect on cell viability (Figure 1c and Supplementary Figure 1B). Calculation of combination index revealed that the interaction of Drozitumab and BV6 is highly synergistic (Table 1). To test the general relevance of the synergistic interaction of BV6 and Drozitumab, we extended our study to pancreatic carcinoma. Similarly, BV6 and Drozitumab cooperated to reduce cell viability in MiaPaCa pancreatic carcinoma cells (Supplementary Figure 1C).

To evaluate the clinical relevance of our approach, we extended our studies to three primary glioblastoma cell cultures and to one glioblastoma stem-like cell line, which were established from distinct surgical samples. Primary glioblastoma cultures have previously been described^20^ and glioblastoma stem-like cells, implicated to contribute to glioblastoma progression due to their increased cell death resistance,^21^ were characterized by Nestin staining and CD133 expression (Supplementary Figures 2A and B). Of note, BV6 acted in concert with Drozitumab to reduce cell viability of primary glioblastoma cultures and glioblastoma stem-like cells (Figure 1d).

To test whether the combination treatment with BV6 and Drozitumab affects long-term survival and clonogenic tumor growth of glioblastoma cells, we performed colony formation assays. Of note, BV6 cooperated with Drozitumab to reduce the clonogenic growth of glioblastoma cells, showing that the combination treatment suppresses long-term survival (Figure 1e).

To evaluate the antitumor activity of BV6 and Drozitumab *in vivo,* we used two different *in vivo* models of glioblastoma. First, we used the chicken chorioallantoic membrane (CAM) model, an established preclinical tumor model, for example, for glioblastoma.^22, 23, 24^ Glioblastoma cells were seeded on the CAM of chicken embryos, allowed to settle and to initiate tumors and were then locally treated with BV6 and/or Drozitumab. Importantly, BV6 and Drozitumab acted in concert to significantly suppress tumor growth of glioblastoma *in vivo*, whereas either drug as single agent had no significant effect on tumor growth (Figure 1f and Supplementary Figure 1D). Second, we used an orthotopic glioblastoma model in nude mice to test the antitumor activity of BV6 and Drozitumab *in vivo*. To this end, U87MG cells were stereotactically implanted into the brain of mice, treated with Drozitumab and/or BV6 and assessed for tumor growth by MR imaging. Importantly, BV6 and Drozitumab cooperated to significantly reduce glioblastoma growth *in vivo* compared with tumors that were treated with either drug alone or with vehicle (Figure 1g).

Together, this set of experiments demonstrates that the Smac mimetic BV6 primes glioblastoma cells including primary glioblastoma cultures and glioblastoma stem-like cells for Drozitumab-mediated cytotoxicity and cooperates with Drozitumab to suppress long-term clonogenic survival and *in vivo* tumor growth.

### BV6 sensitizes glioblastoma cells to Drozitumab-induced, caspase-dependent apoptosis

**Figure 2:**
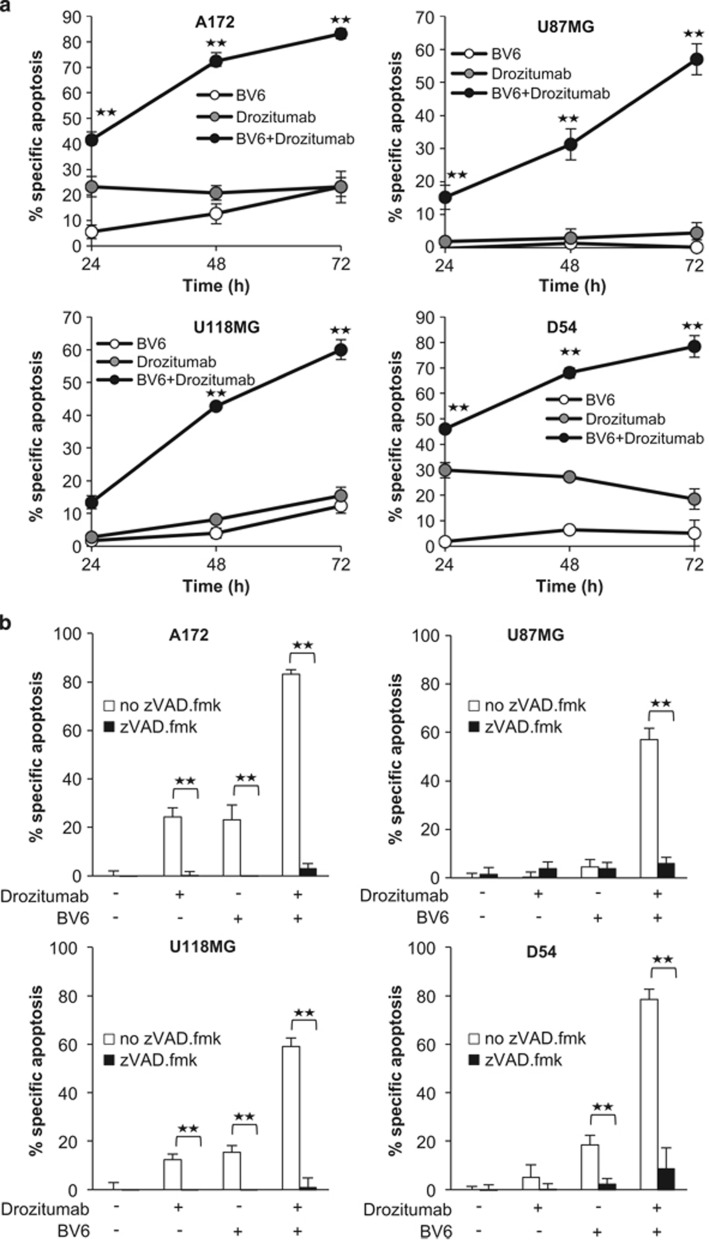

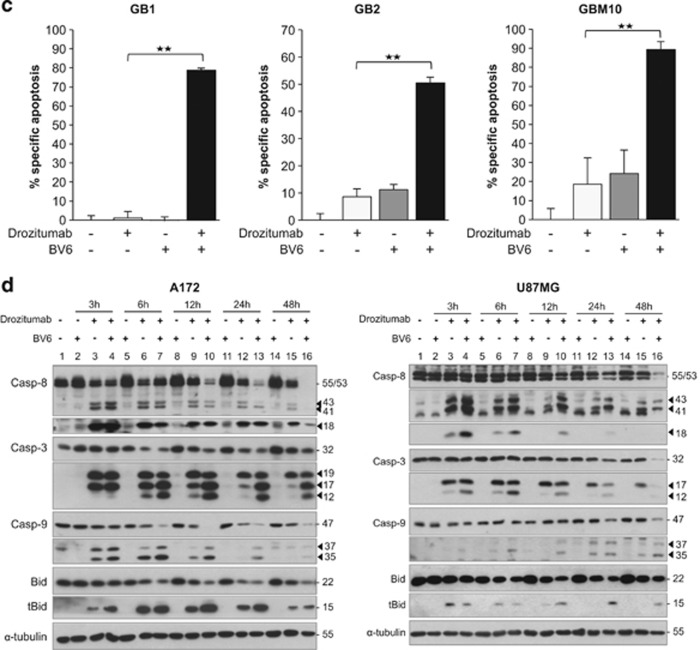
BV6 cooperates with Drozitumab to trigger caspase-dependent apoptosis. (**a**) Glioblastoma cells were treated for 24, 48 and 72 h with Drozitumab (U87MG: 5 *μ*g/ml; A172, D54: 0.3 *μ*g/ml; U118MG: 10 *μ*g/ml) and/or BV6 (U87MG: 4 *μ*M; A172: 3 *μ*M; D54: 2 *μ*M; U118MG: 1 *μ*M). DNA fragmentation of propidium iodide-stained nuclei was determined by FACS analysis. Mean + S.E.M. values of three independent experiments performed in triplicate are shown; **P<0.001. (**b**) Glioblastoma cells were treated for 72 h with Drozitumab (U87MG: 5 *μ*g/ml; A172, D54: 0.3 *μ*g/ml; U118MG: 10 *μ*g/ml) and/or BV6 (U87MG: 4 *μ*M; A172: 3 *μ*M; D54: 2 *μ*M; U118MG: 1 *μ*M) in the presence or absence of 20 *μ*M zVAD.fmk. DNA fragmentation of propidium iodide-stained nuclei was determined by FACS analysis. Mean + S.E.M. values of three independent experiments performed in triplicate are shown; **P<0.001. (**c**) Primary glioblastoma cells (GB1 and GB2) and glioblastoma stem-like cells (GBM10) were treated for 72 h with BV6 and/or Drozitumab (GB1: 8 *μ*M BV6/0.6 *μ*g/ml Drozitumab, GB2: 2 *μ*M BV6/0.6 *μ*g/ml Drozitumab, GBM10: 2.5 *μ*M BV6/10 *μ*g/ml Drozitumab). DNA fragmentation of propidium iodide-stained nuclei was determined by FACS analysis. Mean + S.E.M. values of three independent experiments performed in triplicate are shown; **P<0.001. (**d**) A172 (left panel) and U87MG (right panel) cells were treated for indicated times with 0.3 *μ*g/ml (A172) or 5 *μ*g/ml (U87MG) Drozitumab and/or 3 *μ*M (A172) or 4 *μ*M (U87MG) BV6. Caspase activation and Bid processing were analyzed by western blotting, arrowheads indicate cleavage fragments. A representative experiment of three independent experiments is shown

To investigate whether cells die by apoptotic cell death, we evaluated DNA fragmentation as a characteristic feature of apoptosis. BV6 significantly increased Drozitumab-induced DNA fragmentation in a time-dependent manner in several glioblastoma cell lines (Figure 2a and Supplementary Figure 3A). Addition of the broad-range caspase inhibitor zVAD.fmk as well as caspase-8 or -9 selective inhibitors significantly inhibited DNA fragmentation induced by the combination treatment (Figure 2b and Supplementary Figure 3B), underscoring that cells undergo caspase-dependent apoptosis. Similarly, BV6 and Drozitumab acted together to trigger DNA fragmentation in primary glioblastoma cultures as well as in glioblastoma stem-like cells (Figure 2c).

To gain insights into the molecular mechanisms underlying the cooperative induction of apoptosis by BV6 and Drozitumab, we selected the two glioblastoma cell lines A172 and U87MG. Monitoring of caspase cleavage revealed that BV6 cooperated with Drozitumab to trigger activation of the caspase cascade in glioblastoma cell lines and also in primary glioblastoma stem-like cells (Figure 2d and Supplementary Figure 2C). This was evident, especially at later time points, from the increased cleavage of caspases into active cleavage fragments in cotreated cells compared with cells treated with Drozitumab alone (Figure 2d), that is, p18 caspase-8 fragment (A172: lane 10; U87MG: lane 4), p17/12 caspase-3 fragments (A172: lane 13; U87MG: lane 7) and p37/35 caspase-9 fragments (A172: lane 10; U87MG: lane 10). In addition, proteolytic processing of caspase-8, -3 and -9 in cotreated cells resulted in reduced expression levels of their proenzyme forms (Figure 2d). Moreover, BV6 enhanced Drozitumab-induced cleavage of Bid into the activated form tBid, in particular in A172 cells (Figure 2d). Together, this set of experiments demonstrates that BV6 enhances Drozitumab-mediated caspase activation and caspase-dependent apoptosis in glioblastoma cells.

### cFLIP is a key inhibitor of BV6 and Drozitumab-induced apoptosis

**Figure 3:**
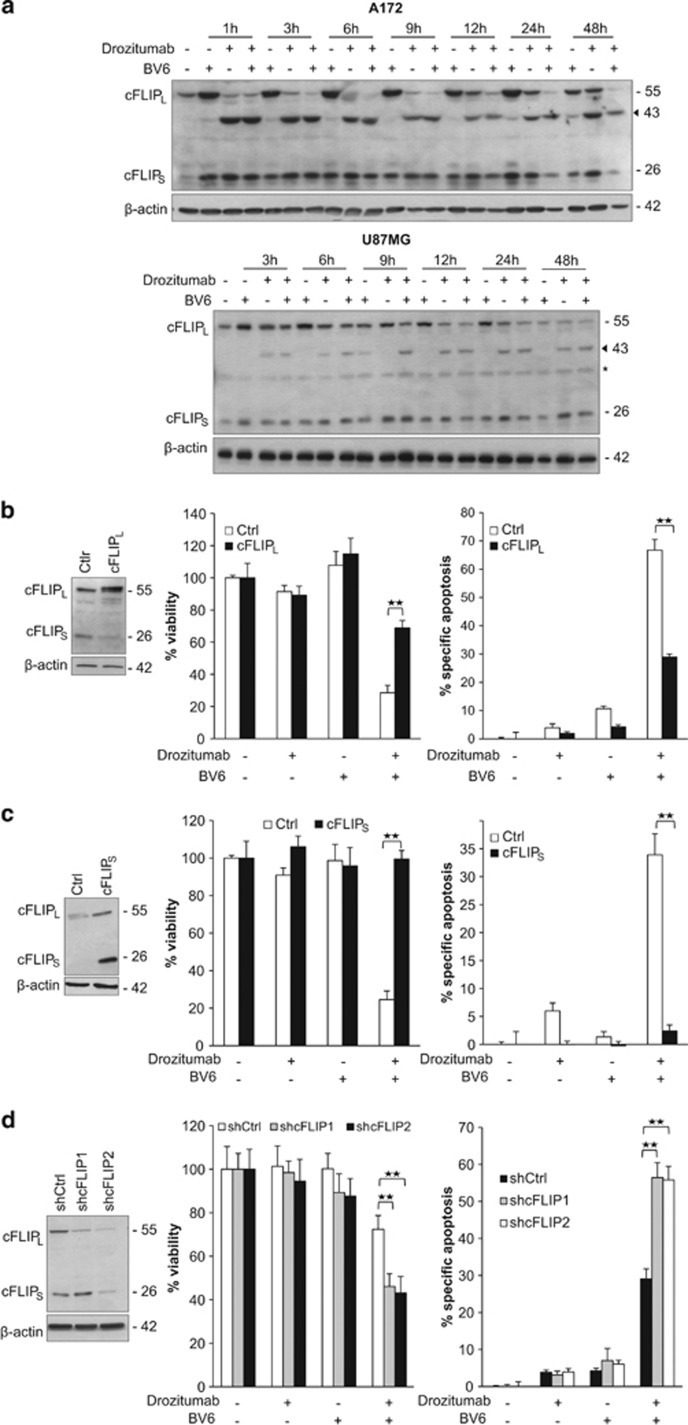
cFLIP is a key inhibitor of BV6- and Drozitumab-induced apoptosis. (**a**) A172 (upper panel) and U87MG (lower panel) cells were treated for indicated times with 0.3 *μ*g/ml (A172) or 5 *μ*g/ml (U87MG) Drozitumab and/or 3 *μ*M (A172) or 4 *μ*M (U87MG) BV6. cFLIP expression levels were analyzed by western blotting, arrowheads indicate cleavage fragments, asterisk indicates unspecific binding. A representative experiment of three independent experiments is shown. (**b**) U87MG cells were transduced with a vector containing cFLIP_L_ or empty vector (Ctrl). Expression of cFLIP was assessed by western blotting (left panel). Cells were treated with 5 *μ*g/ml Drozitumab and/or 4 *μ*M BV6 for 72 h. Cell viability was determined by MTT assay and is expressed as percentage of untreated controls (middle panel). Apoptosis was determined by FACS analysis of DNA fragmentation of propidium iodide-stained nuclei (right panel). Data represent mean + S.E.M. of three independent experiments performed in triplicate; **P<0.001. (**c**) U87MG cells were transduced with a vector containing cFLIP_S_ or empty vector (Ctrl). Expression of cFLIP was assessed by western blotting (left panel). Cells were treated with 5 *μ*g/ml Drozitumab and/or 4 *μ*M BV6 for 72 h. Cell viability was determined by MTT assay and is expressed as the percentage of untreated controls (middle panel). Apoptosis was determined by FACS analysis of DNA fragmentation of propidium iodide-stained nuclei (right panel). Data represent mean + S.E.M. of three independent experiments performed in triplicate; **P<0.001. (**d**) U87MG cells were transduced with control vector (shCtrl) or vectors containing two different shRNA sequences against cFLIP (shcFLIP1, shcFLIP2). Expression of cFLIP was assessed by western blotting (left panel). Cells treated with 0.3 *μ*g/ml Drozitumab and/or 4 *μ*M BV6 for 72 h. Cell viability was determined by MTT assay and is expressed as percentage of untreated controls (middle panel). Apoptosis was determined by FACS analysis of DNA fragmentation of propidium iodide-stained nuclei (right panel). Data represent mean + S.E.M. of three independent experiments performed in triplicate; **P<0.001

As cFLIP is a key regulator of TRAIL- or Smac mimetic-induced apoptosis,^25, 26^ we investigated its expression levels in A172 and U87MG cells. Treatment with Drozitumab or the combination treatment caused rapid and almost complete cleavage of cFLIP_L_ in A172 cells, whereas cleavage of cFLIP_L_ was slower and incomplete in U87MG cells (Figure 3a). Treatment with BV6 alone resulted in the upregulation of both cFLIP isoforms (Figure 3a, upper panel), consistent with the reported BV6-mediated NF-*κ*B activation.^27^ Treatment with Drozitumab alone only transiently caused proteolytic cleavage of cFLIP_L_ into its p43 cleavage fragment in A172 cells, whereas re-expression of cFLIP_L_ occurred after prolonged exposure to Drozitumab (Figure 3a, upper panel). By comparison, the addition of BV6 to Drozitumab resulted in almost complete downregulation of cFLIP_L_ up to 48 h and prevented the re-expression of cFLIP_L_ upon extended treatment in A172 cells (Figure 3a, upper panel). Reduction of cFLIP_S_ protein levels was observed following prolonged combination treatment especially in A172 cells (Figure 3a).

To investigate the role of cFLIP in this model of apoptosis, we selectively upregulated the long and the short isoforms of cFLIP. Ectopic expression of either cFLIP_L_ or cFLIP_S_ in U87MG cells significantly reduced the Smac mimetic-mediated sensitization to Drozitumab-induced loss of cell viability and apoptosis (Figures 3b and c). *Vice versa*, silencing of cFLIP by RNA interference significantly enhanced Drozitumab-induced loss of cell viability and apoptosis (Figure 3d). These data indicate that both cFLIP_L_ and cFLIP_S_ isoforms are negative regulators of BV6/Drozitumab-induced cell death. Cleavage of cFLIP upon cotreatment with BV6 and Drozitumab may contribute to the induction of apoptosis.

### TNF*α* autocrine loop is dispensable for BV6- and Drozitumab-induced apoptosis

**Figure 4:**
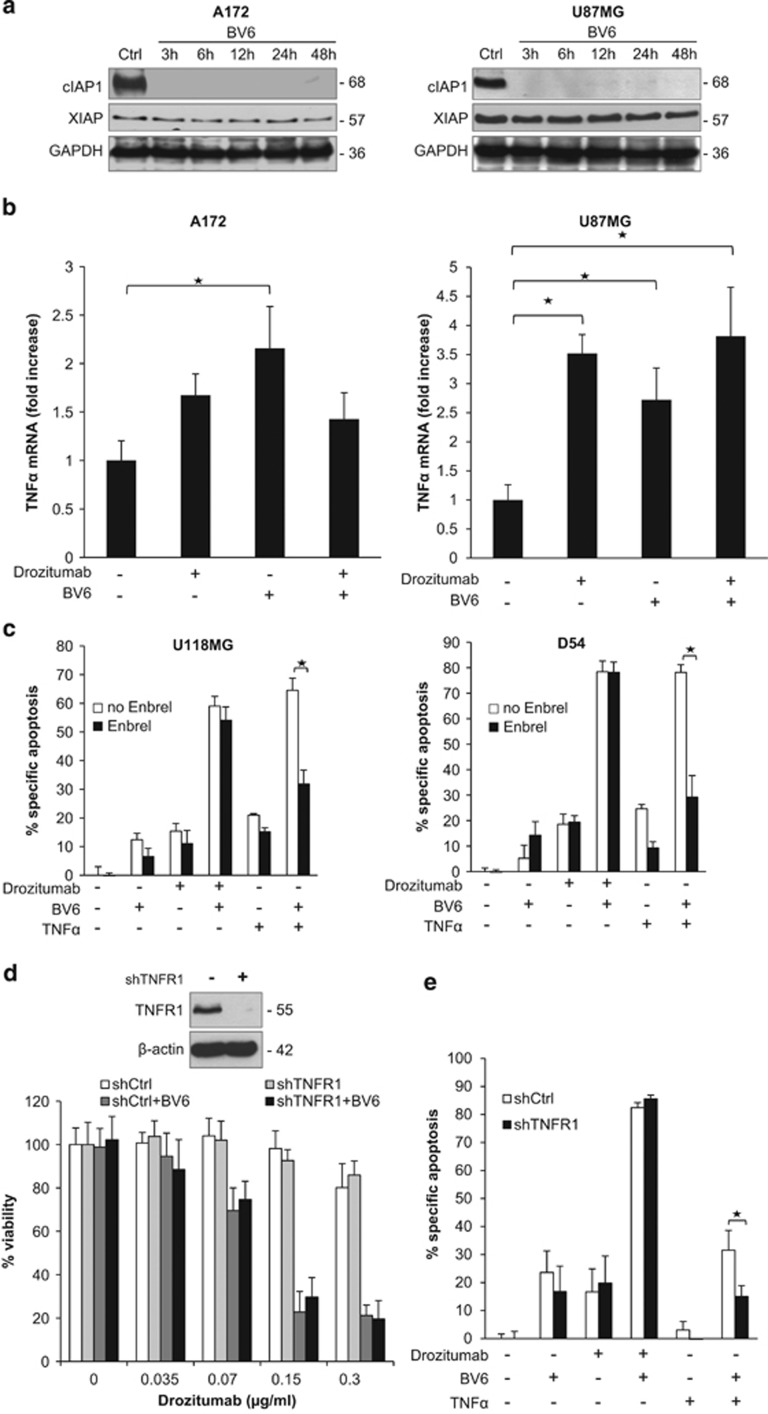
TNF*α* is dispensable for BV6- and Drozitumab-induced apoptosis. (**a**) Glioblastoma cells were treated for indicated times with 3 *μ*M (A172) or 4 *μ*M (U87MG) BV6. Expression of cIAP1 and XIAP was analyzed by western blotting. GAPDH served as loading control. (**b**) Glioblastoma cells were treated for 3 h with 0.3 *μ*g/ml (A172) or 5 *μ*g/ml (U87MG) Drozitumab and/or 3 *μ*M (A172) or 4 *μ*M (U87MG) BV6. TNF*α* mRNA expression was determined by RT-PCR analysis. Fold change of TNF*α* mRNA levels is shown. Data represent mean + S.E.M. of three independent experiments performed in triplicate; *P<0.05. (**c**) Glioblastoma cells were pretreated or not for 1 h with 100 *μ*g/ml Enbrel and then treated for 72 h with Drozitumab at 0.3 *μ*g/ml (D54) or 10 *μ*g/ml (U118MG) and/or BV6 at 1 *μ*M (U118MG) or 2 *μ*M (D54). Apoptosis was determined by FACS analysis of DNA fragmentation of propidium iodide-stained nuclei. Treatment with 5 ng/ml TNF*α* and BV6 for 72 h served as positive control for Enbrel. Data represent mean + S.E.M. of three independent experiments performed in triplicate; *P<0.05. (**d** and **e**) A172 cells were transduced with shRNA vector against TNFR1 (shTNFR1) or control sequence (shCtrl). Expression of TNFR1 was analyzed by western blotting (**d**, upper left panel). A172 cells were treated with indicated concentrations of Drozitumab and/or BV6 for 72 h, cell viability was determined by MTT assay and is expressed as percentage of untreated controls (**d**, lower left panel). A172 cells were treated with indicated concentrations of Drozitumab and/or BV6 for 72 h and apoptosis was determined by FACS analysis of DNA fragmentation of propidium iodide-stained nuclei (**e**). Treatment with 5 ng/ml TNF*α* and 3 *μ*M BV6 for 72 h served as positive control for TNFR1 knockdown. In **d** and **e**, data represent mean + S.E.M. of three independent experiments performed in triplicate; *P<0.05

Smac mimetics have been reported to trigger autoubiquitination and proteasomal degradation of cIAP proteins leading to NF-*κ*B activation.^9, 10, 14^ Therefore, we next analyzed the effect of BV6 on the expression levels of IAP proteins. Figure 4a shows that BV6 caused rapid downregulation of cIAP1, whereas XIAP levels only slightly decreased after longer exposure. To investigate the involvement of NF-*κ*B in BV6/Drozitumab-induced cell death, we used glioblastoma cells that overexpress I*κ*B*α* superrepressor (I*κ*B*α*-SR).^27^ NF-*κ*B inhibition protected against BV6/Drozitumab-induced loss of cell viability (Supplementary Figure 4A), demonstrating that NF-*κ*B is required for BV6/Drozitumab-induced cell death.

Smac mimetic-triggered apoptosis has been described to depend on an autocrine TNF*α* loop that leads to activation of caspase-8 and subsequently to apoptosis.^9, 10, 14^ Therefore, we analyzed the production of TNF*α*. Treatment with BV6 and/or Drozitumab resulted in increased mRNA levels of TNF*α* (Figure 4b). Next, we assessed the role of TNF*α* in BV6- and Drozitumab-mediated apoptosis using both a pharmacological and a genetic approach. The addition of Enbrel, a soluble TNF*α*-blocking antibody, did not inhibit the combination treatment-induced apoptosis in several glioblastoma cell lines, whereas Enbrel blocked apoptosis upon treatment with BV6 and TNF*α*, which was used as a positive control (Figure 4c). Also, Enbrel failed to interfere with BV6/Drozitumab-triggered loss of viability (Supplementary Figure 4B). Similarly, the knockdown of TNFR1 had no effect on cell viability or apoptosis of cells treated with the combination of BV6 and Drozitumab, whereas it significantly reduced BV6 plus TNF*α*-induced apoptosis (Figures 4d and e). Together, these results indicate that the synergistic induction of apoptosis by BV6 and Drozitumab does not require TNF*α*-mediated signaling.

### RIP1 is required for Smac mimetic- and Drozitumab-induced apoptosis

**Figure 5:**
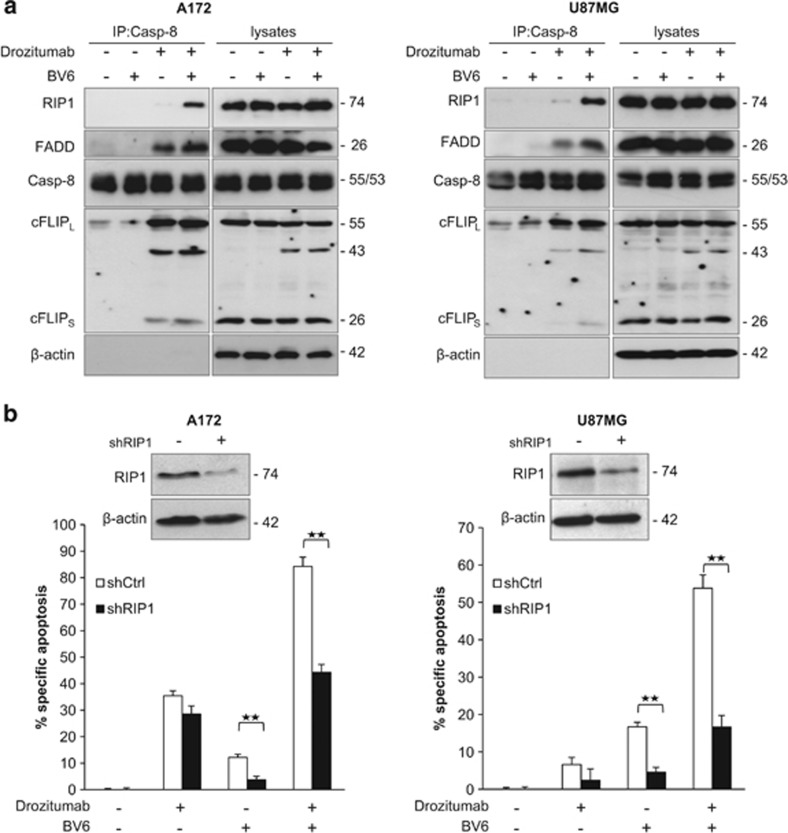

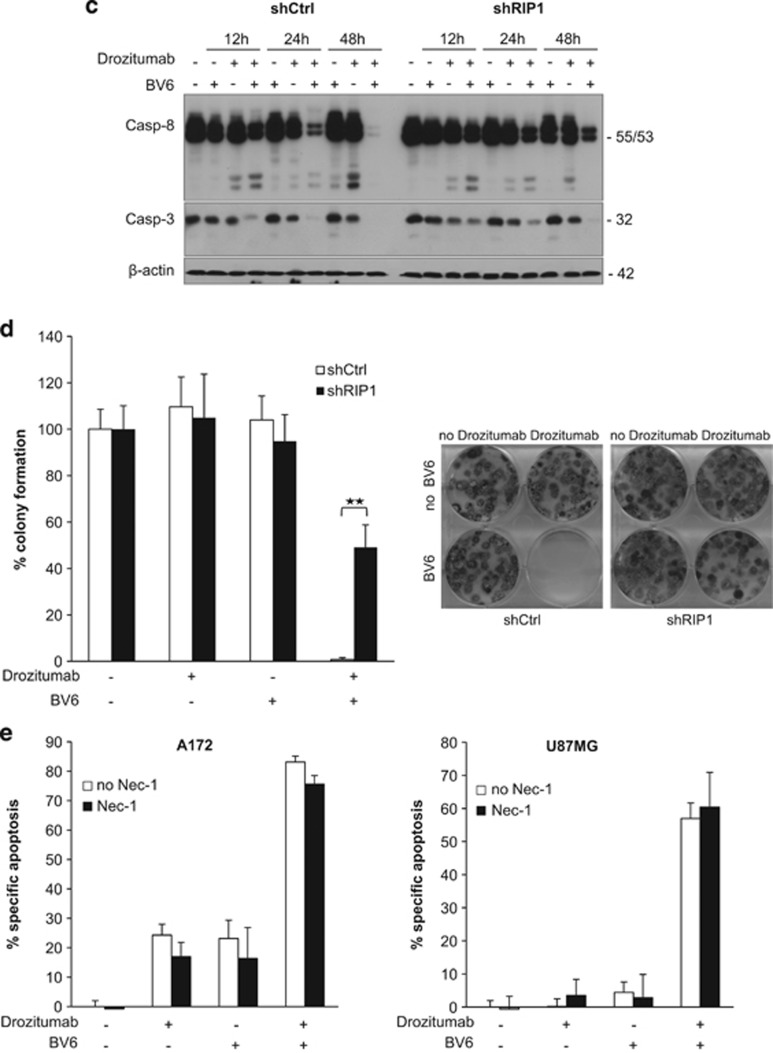
RIP1 is required for BV6- and Drozitumab-induced apoptosis. (**a**) A172 (left panel) and U87MG (right panel) cells were treated for 1 h with 0.3 *μ*g/ml (A172) or 5 *μ*g/ml (U87MG) Drozitumab and/or 3 *μ*M (A172) or 4 *μ*M (U87MG) BV6. Caspase-8 was immunoprecipitated (IP) using an anti-caspase-8 antibody and indicated proteins were detected by western blot analysis. A representative experiment of three independent experiments is shown. (**b**–**d**) A172 (left panels) and U87MG (right panels) cells were transduced with shRNA vector against RIP1 (shRIP1) or control vector (shCtrl). Expression of RIP1 was analyzed by western blotting (**b**, upper parts). Cells were treated for 72 h with 0.3 *μ*g/ml (A172) or 5 *μ*g/ml (U87MG) Drozitumab and/or 3 *μ*M (A172) or 4 *μ*M (U87MG) BV6 and apoptosis was determined by FACS analysis of DNA fragmentation of propidium iodide-stained nuclei (**b**, lower parts). In (**c**), A172 cells with RIP1 knockdown or control vector cells were treated for indicated times with 0.3 *μ*g/ml Drozitumab and/or 3 *μ*M BV6 and caspase activation was analyzed by western blotting. A representative experiment of two independent experiments is shown. (**d**) U87MG cells with RIP1 knockdown or control vector cells were treated for 72 h with 5 *μ*g/ml Drozitumab and/or 4 *μ*M BV6 and then seeded as single cells. Colony formation was assessed by crystal violet staining after 14 days and colonies were counted under the microscope. One representative experiment of three independent experiments (right panel) and the percentage of colony numbers compared with untreated control with mean + S.E.M. of three independent experiments performed in triplicate (**P<0.001) are shown (left panel). (**e**) A172 (left panel) and U87MG (right panel) cells were treated for 72 h with 0.3 *μ*g/ml (A172) or 5 *μ*g/ml (U87MG) Drozitumab and/or 3 *μ*M (A172) or 4 *μ*M (U87MG) BV6 in the presence or absence of 30 *μ*M necrostatin-1 (Nec-1). Apoptosis was determined by FACS analysis of DNA fragmentation of propidium iodide-stained nuclei. Mean + S.E.M. of three independent experiments performed in triplicate are shown

As the combination treatment cooperated to induce caspase-8 activation (Figure 2d), we next analyzed DISC formation, one of the earliest events in the TRAIL signaling pathway. However, the addition of BV6 had no detectable effect on the recruitment of FADD and caspase-8 to activated TRAIL receptors (Supplementary Figure 5A). As the degradation of cIAPs has been reported to result in deubiquitination of RIP1, thereby promoting the formation of a cytosolic caspase-8-activating complex together with FADD and RIP1,^12, 13^ we next examined the assembly of this complex. Indeed, we found that BV6 plus Drozitumab cooperated to trigger formation of the complex containing RIP1, caspase-8 and FADD compared with treatment with Drozitumab alone (Figure 5a). Depletion of lysates from the TRAIL DISC complex before immunoprecipitation confirmed that BV6/Drozitumab cotreatment induced the assembly of a cytosolic RIP1/FADD/caspase-8 complex (Supplementary Figure 5A). To examine the requirement of RIP1 in this model of apoptosis, we used both genetic and pharmacological approaches to inhibit RIP1. Strikingly, knockdown of RIP1 by RNA interference markedly reduced the BV6-mediated sensitization towards Drozitumab-induced DNA fragmentation, caspase activation and loss of clonogenic survival (Figures 5b–d). Similarly, silencing of RIP1 by an independent sequence significantly inhibited BV6- and Drozitumab-induced apoptosis and loss of cell viability (Supplementary Figure 5B). In a second approach, we used necrostatin-1, a selective inhibitor of RIP1 kinase activity.^28^ Necrostatin-1 rescued neither the combination treatment-induced DNA fragmentation nor loss of viability (Figure 5e and Supplementary Figures 5C and D). By comparison, necrostatin-1 significantly reduced BV6 and TNF*α*-induced necroptotic cell death in FADD-deficient Jurkat cells that were used as positive control (Supplementary Figure 5E). Consistently, no RIP3 protein expression was detected in glioblastoma cells in comparison with Jurkat cells used as a positive control (Supplementary Figure 5F). These experiments using distinct targeting sequences to silence RIP1 indicate that RIP1 protein is required for BV6/Drozitumab-induced apoptosis, while RIP1 kinase activity is largely dispensable for cell death induction.

## Discussion

In this study, we present a preclinical evaluation of a rational combination of two novel compounds for the treatment of glioblastoma, that is, the Smac mimetic BV6, which antagonizes IAP proteins, and Drozitumab, a monoclonal antibody that specifically targets TRAIL-R2. The study is built on the strong mechanism-based hypothesis that neutralizing IAP proteins by Smac mimetic lowers the threshold for apoptosis induction and potentiates the antitumor activity of TRAIL receptor agonists.

Here, we report that the Smac mimetic BV6 synergizes with the TRAIL-R2-specific antibody Drozitumab to trigger apoptosis in glioblastoma cells. The potency of this combination is demonstrated by statistical analysis of synergistic effects with a high degree of synergism (combination index<0.1). Synergistic induction of apoptosis by BV6 and Drozitumab is presented in several glioblastoma cell lines confirming the generality of these results. Data obtained in glioblastoma cell lines are supported by parallel experiments using clinical tumor material derived from patients with glioblastoma and two *in vivo* models of glioblastoma including an orthotopic mouse model, thus underscoring the clinical relevance of this combination strategy. Together, these findings indicate that BV6 represents a promising new approach to prime glioblastoma cells towards Drozitumab, which warrants further investigation.

We identify RIP1 as a critical mediator of the synergistic induction of apoptosis that is required for BV6- and Drozitumab-triggered apoptosis, because knockdown of RIP1 significantly decreases apoptosis. BV6 and Drozitumab act together to stimulate the assembly of a cytosolic complex containing RIP1/FADD/caspase-8 that drives caspase-8 activation. By comparison, the pharmacological inhibition of RIP1 kinase activity by necrostatin-1 has little effect on the combination treatment-induced formation of the RIP1/FADD/caspase-8 complex and apoptosis. This indicates that RIP1 may function as an important scaffold protein that promotes the assembly of the RIP1/FADD/caspase-8 complex, whereas RIP1 kinase activity is dispensable in this model of apoptosis. Consistent with a previous study,^29^ autocrine TNF*α* signaling is not involved in BV6/Drozitumab-mediated apoptosis, as pharmacological or genetic inhibition of TNF*α*/TNFR1 signaling by the TNF*α*-blocking antibody Enbrel or by RNA interference-mediated silencing of TNFR1 failed to prevent BV6/Drozitumab-induced apoptosis. Thus, the current study demonstrates for the first time that Smac mimetic BV6 and Drozitumab synergize to trigger apoptosis in a RIP1-dependent, but TNF*α*-independent manner in glioblastoma cells.

Previously, an autocrine TNF*α* loop has been implicated to mediate apoptosis upon single agent treatment with cytotoxic concentrations of Smac mimetics that stimulate the production of TNF*α*, thereby initiating TNFR1-mediated cell death.^9, 10, 14^ In the present study, however, BV6 at subtoxic concentrations acts together with Drozitumab to trigger the RIP1-containing cell death complex and apoptosis independently of TNF*α*. We previously reported that CD95-blocking antibodies failed to rescue BV6- and TMZ-induced apoptosis.^27^ This indicates that Smac mimetics can cooperate with different death receptor ligands, that is, TNF*α* and TRAIL receptor agonists, to promote the assembly of the RIP1/FADD/caspase-8 complex.

RIP1 is emerging as a key regulator of both apoptotic and necroptotic cell death. For example, RIP1-containing multimeric complexes have been described in the context of genotoxic damage or toll-like receptor-3 activation and were shown to mediate apoptosis in a TNF*α*-dependent or -independent manner.^27, 30, 31, 32, 33^ Furthermore, RIP1 was identified as a crucial regulator of necroptosis in a genome-wide RNA interference screen^34^ and subsequently shown to be required for the formation of the RIP1/RIP3 necrosome complex.^35, 36^ Also, a mixed type of cell death with both apoptotic and necrotic features has been reported upon loss of cIAP proteins that was inhibited by a combination of RIP1 kinase and caspase inhibitors.^37^ Thus, whether RIP1 engages apoptotic or necroptotic signaling cascades and whether these RIP1-mediated signaling events occur in a TNF*α*-dependent or -independent manner is contingent on the specific context, that is, the cell type and/or stimulus. The findings of our present study showing several characteristic features of apoptosis including DNA fragmentation, caspase activation and sensitivity to the caspase inhibitor zVAD.fmk support the notion that the combination of BV6 and Drozitumab triggers apoptosis.

In this model of BV6/Drozitumab-induced apoptosis, both cFLIP_L_ and cFLIP_S_ exert an anti-apoptotic function. This finding is in line with previous reports showing that cFLIP is a negative regulator of cell death induced by Smac mimetic or genotoxic drugs that cause depletion of IAP proteins.^25, 31^ By comparison, cFLIP_L_ and cFLIP_S_ have recently been reported to differentially regulate cell death in the absence of cIAPs in keratinocytes.^32^ Whereas cFLIP_L_ blocked RIP1/FADD/caspase-8 complex formation and cell death, cFLIP_S_ promoted these events.^32^ These results point to a context-dependent role of cFLIP_L_ and cFLIP_S_ in the regulation of IAP inhibitor-mediated cell death.

Together, our findings provide novel insights into the synergistic action of Smac mimetic and TRAIL receptor agonists compared with previous studies in other cancers. Indeed, owing to the lack of differences in caspase-8 activation between Drozitumab alone or in combination with BV6, Varfolomeev *et al.*^29^ accounted in the ability of BV6 to inhibit XIAP-mediated inhibition of effector caspases the main determinant of the pro-apoptotic enhancement of Drozitumab-induced apoptosis. In our study, instead, we identify RIP1 as a key mediator of BV6/Drozitumab-triggered apoptosis, whereas the sole inhibition of XIAP activity by BV6 would not be sufficient to explain the marked increase in caspase-8 activation observed in cotreated cells.

Our findings have several important implications. First, our study provides a rational basis for the future (pre)clinical development of Smac mimetics such as BV6 and TRAIL receptor agonists such as Drozitumab in glioblastoma. The clinical relevance of our findings is underscored by the confirmation of the synergistic induction of apoptosis by BV6/Drozitumab in primary glioblastoma cultures isolated from tumor specimens. Further, TRAIL-R2-targeting agents are of particular interest in glioblastoma because of frequent epigenetic inactivation of TRAIL-R1 in this tumor.^18^ As Smac mimetics as well as proapoptotic TRAIL receptor agonists are currently under evaluation in early clinical trials,^16, 38^ it may in principle be feasible to transfer this combination approach to the clinical stage. Second, our data provide novel insights into the mechanisms that regulate signal transduction events upon the combination treatment with Smac mimetics and TRAIL receptor agonists. These results have important implications also for other cancers beyond glioblastoma, underscoring the general relevance of this study.

In conclusion, this study provides convincing evidence showing that the Smac mimetic BV6 in combination with the TRAIL-R2 agonist Drozitumab presents a promising strategy to trigger apoptosis pathways in glioblastoma, which warrants further investigation.

## Materials and Methods

### Cell culture and chemicals

Glioblastoma cell lines were obtained from the American Type Culture Collection (Manassas, VA, USA) and maintained in DMEM medium (Life Technologies, Inc., Eggenstein, Germany), supplemented with 10 % fetal calf serum (Biochrom, Berlin, Germany), 1 mM glutamine (Invitrogen, Karlsruhe, Germany), 1 % penicillin/streptomycin (Invitrogen) and 25 mM HEPES (Biochrom) as described previously.^20^ U87MG cells stably expressing cFLIP_L_ or cFLIP_S_ or with stable knockdown of cFLIP have previously been described^39^ as well as A172 cells with stable knockdown of TNFR1.^27^ Primary glioblastoma cultures (GB1-GB3) were established from surgical specimens obtained from three patients with grade 4 glioma and were cultured as described previously.^20^ Briefly, primary glioblastoma cells were isolated by mechanical disaggregation from surgical specimens and cultured in DMEM supplemented with 10% fetal calf serum (Biochrom), 1 mM glutamine (Biochrom), 1% penicillin/streptavidin (Biochrom) and 25 mM HEPES (Biochrom). The study was approved by the local Ethics Committee. Purity of cultured glioblastoma cells was >90% as assessed by expression of microtubule-associated protein 2. Cells used for this study were between passage 2 and 12. Glioblastoma stem-like cells were established from a surgical specimen of a 70-year-old woman diagnosed with glioblastoma. Immediately after resection, the tumor tissue was minced and dissociated in papain and DNase for 20 min followed by gentle tituration. Cells were then pipetted through a cell strainer (40 *μ*m) to remove larger tissue fragments, washed and resuspended in DMEM/F12 medium (Life Technologies, Inc.) supplemented with B-27 supplement (Invitrogen), 20 ng/ml epidermal growth factor (EGF, R&D Systems, Inc., Wiesbaden, Germany) and 10 ng/ml basic fibroblast growth factor (Sigma, Deisenhofen, Germany). Cells used for this study were between passage 20 and 24 and were characterized by Nestin and CD133 staining. N-benzyloxycarbonyl-Val-Ala-Asp-fluoromethylketone (zVAD.fmk) was purchased from Bachem (Heidelberg, Germany), recombinant human TNF*α* from Biochrom, necrostatin-1 from Biomol (Hamburg, Germany) and all chemicals from Sigma unless indicated otherwise. Enbrel was kindly provided by Pfizer (Berlin, Germany). The fully human agonist monoclonal TRAIL-R2 antibody Drozitumab^19^ and the bivalent Smac mimetic BV6 that potently antagonizes XIAP, cIAP1 and cIAP2^9^ were kind gifts from Genentech (South San Francisco, CA, USA).

### RNA interference

For stable gene knockdown, shRNA targeting RIP1 sequence (CCACTAGTCTGACGGATAA) or a control sequence with no corresponding part in the human genome (gatcatgtagatacgctca) were cloned into pGreenPuro and lentivirus-containing supernatants were generated as described previously.^33^ Stable cell lines were produced by selection with 1 *μ*g/ml puromycin (BD Biosciences, Heidelberg, Germany).

### Determination of apoptosis, cell viability and colony formation

Apoptosis was determined by fluorescence-activated cell-sorting (FACSCanto II, BD Biosciences) analysis of DNA fragmentation of propidium iodide-stained nuclei as described previously.^40^ The percentage of specific apoptosis was calculated as follows: 100 × [experimental apoptosis(%)-spontaneous apoptosis (%)]/[100%-spontaneous apoptosis (%)]. Cell viability was assessed by 3-(4,5-dimethylthiazol-2-yl)-2,5-diphenyltetrazolium bromide (MTT) assay according to the manufacturer's instructions (Roche Diagnostics, Mannheim, Germany). For colony assay, cells were seeded after 72 h of treatment as single cells (200 cells/well) in 6-well plates and colony formation was assessed after additional 14 days by crystal violet staining (0.75% crystal violet, 50% ethanol, 0.25% NaCl, 1.57% formaldehyde).

### Western blot analysis

Western blot analysis was performed as described previously^40^ using the following antibodies: mouse anti-caspase-8, mouse anti-cFLIP (Alexis Biochemicals, Grünberg, Germany), mouse anti-FADD, mouse anti-XIAP (clone 28), mouse anti-RIP1 (BD Transduction Laboratories, Heidelberg, Germany), rabbit anti-Bid, rabbit anti-caspase-3, mouse anti-caspase-9 (Cell Signaling, Beverly, MA, USA), rabbit anti-TRAIL-R2 (Chemicon, Billerica, MA, USA), goat anti-cIAP1 (R&D Systems, Wiesbaden, Germany), rabbit anti-cIAP2 (Epitomics, Burlingname, CA, USA), mouse anti-TNFR1 (Santa Cruz Biotechnology, Santa Cruz, CA, USA). Mouse anti-*α*-tubulin (Calbiochem, Darmstadt, Germany) or mouse anti-*β*-actin (Sigma) were used as loading controls. Goat anti-mouse IgG, donkey anti-goat IgG, goat anti-rabbit IgG conjugated to horseradish peroxidase (Santa Cruz Biotechnology) and goat anti-mouse IgG1 or goat anti-mouse IgG2b (Southern Biotech, Birmingham, AL, USA) conjugated to horseradish peroxidase were used as secondary antibodies. Enhanced chemiluminescence was used for detection (Amersham Bioscience, Freiburg, Germany). Representative blots of at least two independent experiments are shown.

### Immunoprecipitation

Immunoprecipitation of caspase-8 was performed as described previously.^33^ Briefly, cells were lysed in NP40 buffer (10 mM Tris pH 8.0, 150 mM NaCl, 1% NP-40, supplemented with a protease inhibitor tablet (Roche, Grenzach, Germany). One microgram of protein was incubated with 10 *μ*g mouse anti-caspase-8 antibody (Alexis) overnight at 4 °C followed by the addition of 20 *μ*l pan-mouse IgG Dynabeads (Invitrogen), then incubated for 2 h at 4 °C and washed with NP40 buffer. Caspase-8 was detected using rabbit monoclonal anti-caspase-8 antibody (Epitomics), RIP1 or FADD with anti-mouse RIP antibody or mouse-anti-FADD antibody (BD Biosciences).

### Cell surface staining

To determine surface expression of TRAIL receptors, cells were incubated with mouse anti-TRAIL-R1 and -R2 antibodies (10 *μ*g/ml; all from Alexis) for 30 min at 4 °C, washed in PBS containing 1% fetal calf serum, incubated with rabbit anti-mouse F(ab')_2_ IgG/biotin (5 *μ*g/ml; BD Biosciences) for 20 min at 4 °C in the dark, washed in PBS containing 1 % fetal calf serum, incubated with streptavidin-PE (0.25 *μ*g/ml; BD Bioscience) for 20 min at 4 °C in the dark and analyzed by flow cytometry.

### Real-Time PCR analysis

Total RNA was extracted using peqGOLD Total RNA kit from Peqlab Biotechnologie GmbH (Erlangen, Germany) according to the manufacturer's instructions. Two micrograms of total RNA were used for cDNA synthesis using RevertAid H Minus First Strand cDNA Synthesis Kit (Thermo Scientific, Schwerte, Germany). mRNA expression levels were analyzed by quantitative RT-PCR in SYBR Green using the 7900HT fast real-time PCR system from Applied Biosystem (Darmstadt, Germany). Ribosomal 18S was used as a reference gene. The following primers were used: TNF*α* (fwd:ACAACCCTCAGACGCCACAT and rvs:TCCTTTCCAGGGGAGAGAGG) and h18S (fwd: CGCAAATTACCCACTCCCG and rvs: TTCCAATTACAGGGCCTCGAA). PCR was carried out at an annealing temperature of 61 °C. All determinations were performed in triplicate.

### Chorioallantoic membrane (CAM) model

Experiments using the CAM model were done as described previously.^22, 23^ Briefly, 0.8 × 10^6^ cells were implanted on fertilized chicken eggs on day 8 of incubation and were treated with BV6 and/or Drozitumab for 2 days. Tumors were sampled with the surrounding CAM on day 4, fixed in 4% paraformaldehyde, embedded in paraffin, cut in 5 *μ*m sections and were analyzed by immunohistochemistry using 1 : 1 hematoxylin and 0.5% eosin. Images were digitally recorded at × 4 magnification with an SZ61 microscope (Olympus, Center Valley, PA, USA); tumor areas were analyzed with ImageJ digital imaging software (NIH, Bethesda, MA, USA).

### *In vivo* orthotopic glioblastoma mouse model

For the orthotopic glioblastoma model, 1.5 × 10^5^ U87MG cells were implanted stereotactically (1.5 mm dorsal and 1.5 mm ventral to bregma) into the brain of CD1 nude mice (Charles River Laboratories, Sulzfeld, Germany). Animals were anesthetized with a mixture of 0.5 mg/kg Medetomidin, 5.0 mg/kg Midazolam and 0.05 mg/kg Fentanyl; anesthesia was abrogated by injection of 0.5 mg/kg Flumazenil and 2.5 mg/kg Atipamezole. At days 6 and 9 post implantation of tumor cells, 10 *μ*g Drozitumab, 16 nmol BV6, a combination of both compounds or PBS as vehicle were stereotactically injected into tumors in a volume of 2 *μ*l at the same coordinates used for tumor cell implantation. On day 13 post implantation of tumor cells, animals were anesthetized with a mixture of 1.5% isoflurane (Abbott, Wiesbaden, Germany) evaporated in oxygen at a flow of 0.5 l/min. Subsequently, animals were placed in a 1 T Icon-Scanner (Bruker, Ettlingen, Germany) equipped with a mouse brain coil and T2-weighted images of the brain were acquired. Body temperature was maintained at 37 °C by a heating system and a rectal temperature sensor. Tumor volumes were determined by manually drawing regions of interest in the MR images in Inveon Research Workplace 3.1 (Siemens Preclinical Solutions, Erlangen, Germany) and creating volumes of interest. As the experiments were performed with two different starting days, the mean tumor volume of the vehicle group of each starting day was set as 100% and the tumor volumes of all animals with the same starting day were related to this.

### Statistical analysis

Statistical significance was assessed by Student's *t*-Test (two-tailed distribution, two-sample, unequal variance). Interaction between IAP inhibitors and Drozitumab was analyzed by the Combination Index method based on that described by Chou^41^ using CalcuSyn software (Biosoft, Cambridge, UK). Combination index<0.9 indicates synergism, 0.9–1.1 additivity and > 1.1 antagonism.

## Figures and Tables

**Table 1:** Drozitumab and BV6 synergistically induce apoptosis in several glioblastoma cell lines

**U87MG Drozitumab (*μ*g/ml)**	**BV6 (*μ*M)**	**CI**	**T98G Drozitumab (*μ*g/ml)**	**BV6 (*μ*M)**	**CI**	**A172 Drozitumab (*μ*g/ml)**	**BV6 (*μ*M)**	**CI**	**U118MG Drozitumab (*μ*g/ml)**	**BV6 (*μ*M)**	**CI**	**U138MG Drozitumab (*μ*g/ml)**	**BV6 (*μ*M)**	**CI**
1.25	2	0.107	0.15	2	0.235	0.3	2	0.404	1.25	1	0.083	1.25	2	0.947
1.25	3	0.109	0.15	3	0.326	0.3	3	0.619	1.25	2	0.146	1.25	3	0.734
1.25	4	0.111	0.15	4	0.346	0.3	4	0.773	1.25	3	0.204	1.25	4	0.879
2.5	2	0.079	0.3	2	0.144	0.6	2	0.426	2.5	1	0.089	2.5	2	0.861
2.5	3	0.090	0.3	3	0.206	0.6	3	0.625	2.5	2	0.134	2.5	3	0.612
2.5	4	0.094	0.3	4	0.260	0.6	4	0.796	2.5	3	0.139	2.5	4	0.804
5	2	0.073	0.6	2	0.089	1.25	2	0.481	5	1	0.084	5	2	0.597
5	3	0.083	0.6	3	0.141	1.25	3	0.685	5	2	0.110	5	3	0.531
5	4	0.089	0.6	4	0.146	1.25	4	0.857	5	3	0.149	5	4	0.699
10	2	0.071	1.25	2	0.081	2.5	2	0.566	10	1	0.075	10	2	0.556
10	3	0.084	1.25	3	0.158	2.5	3	0.789	10	2	0.122	10	3	0.501
10	4	0.104	1.25	4	0.145	2.5	4	0.950	10	3	0.131	10	4	0.717

Combination index (CI) was calculated as described by Chou^41^ using CalcuSyn software (Biosoft, Cambridge, UK) for loss of cell viability induced by combined treatment of cells for 72 h with indicated concentrations of Drozitumab and BV6. Cell viability was determined by MTT assay. CI <0.9 indicates synergism, 0.9–1.1 additivity and >1.1 antagonism

## Abbreviations

CAM: chicken chorioallantoic membrane
CI: combination Index
cIAP: cellular Inhibitor of Apoptosis
DISC: death-inducing signaling complex
IAP: Inhibitor of Apoptosis
I*κ*B*α*-SR: I*κ*B*α* superrepressor
MTT: 3-(4,5-dimethylthiazol-2-yl)-2,5-diphenyltetrazolium bromide
RIP1: receptor-activating protein 1
Smac: second mitochondria-derived activator of caspases
TNF: tumor necrosis factor
zVAD.fmk: N-benzyloxycarbonyl-Val-Ala-Asp-fluoromethylketone
