# Supplementary material for: Identification of RIP1 as a critical mediator of Smac mimetic-mediated sensitization of glioblastoma cells for Drozitumab-induced apoptosis
Source: Cell Death Dis. 2015 Apr 16;6(4):e1724–. doi: 10.1038/cddis.2014.592 (PMC4650534; doi:10.1038/cddis.2014.592)
Supplement: Supplementary Information [file cddis2014592x1.pdf]

Suppl. Tab. 1

	**p53 status**	**PTEN status**
**U87MG**	wt	mutated
**T98G**	mutated	wt
**A172**	wt	deleted
**U118MG**	mutated	mutated
**U138MG**	mutated	deleted
**D54**	wt	mutated
**LN229**	wt	wt

Suppl.Fig. 1

A

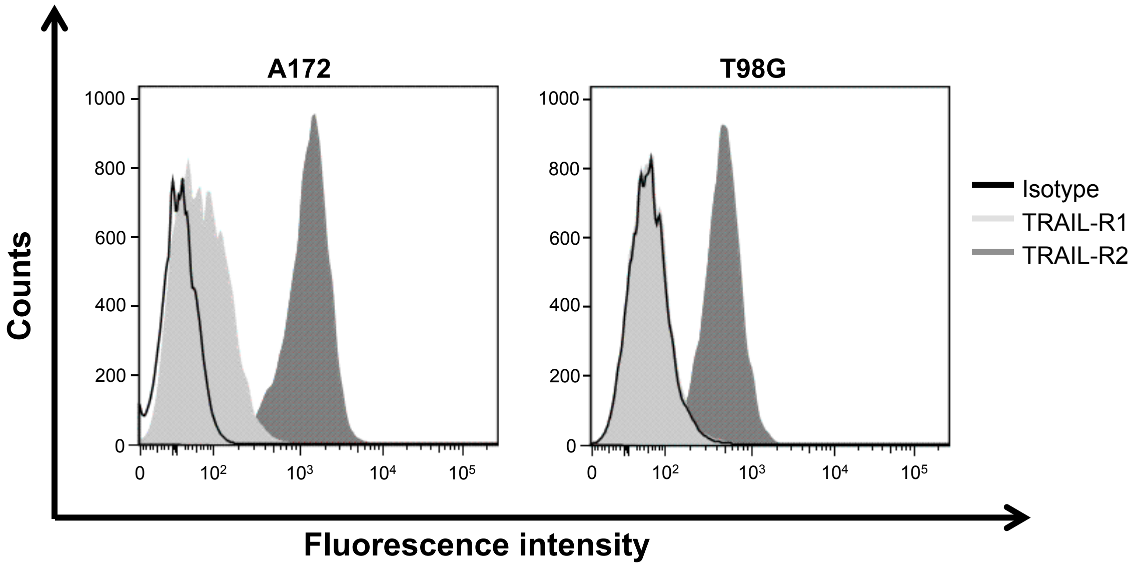

B

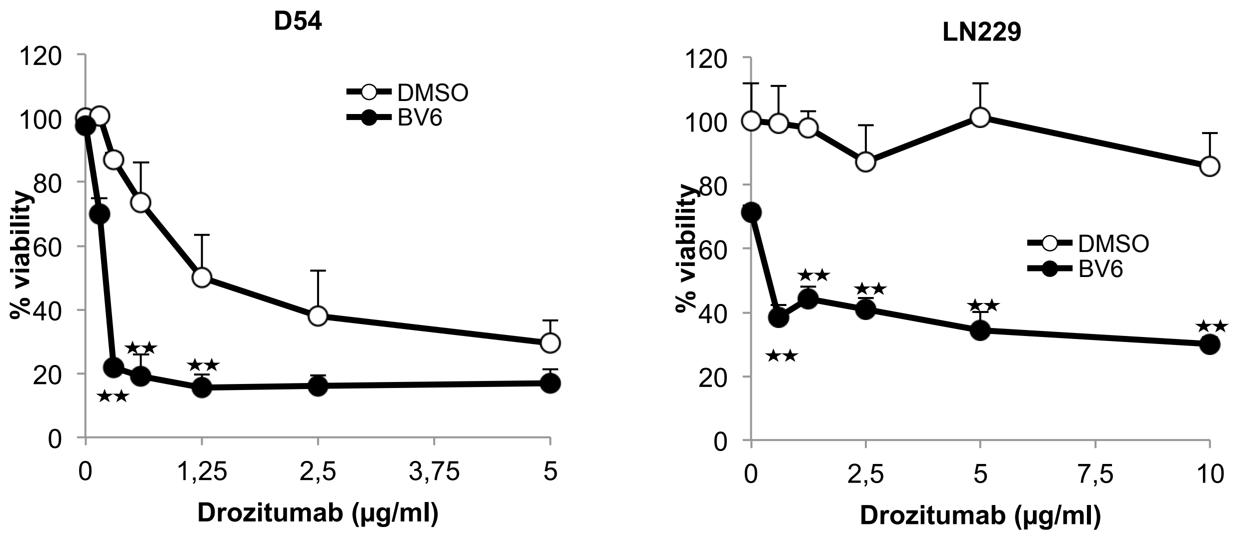

C

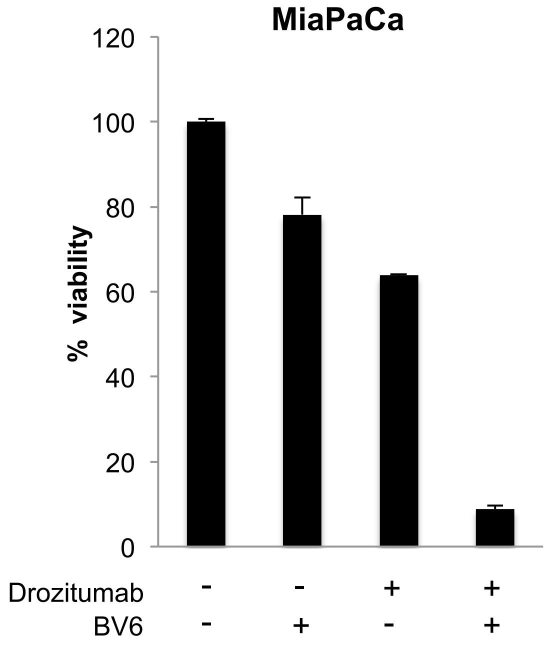
MiaPaCa
120
100
80
60
40
20
0
% viability
Drozitumab - - + +
BV6 - + - +

D

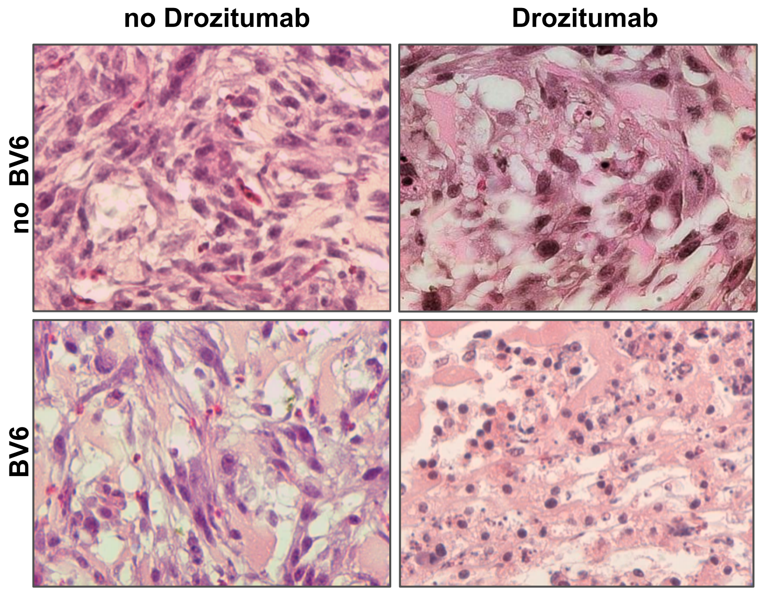
no Drozitumab
Drozitumab
no BV6
BV6

Suppl.Fig. 2

A

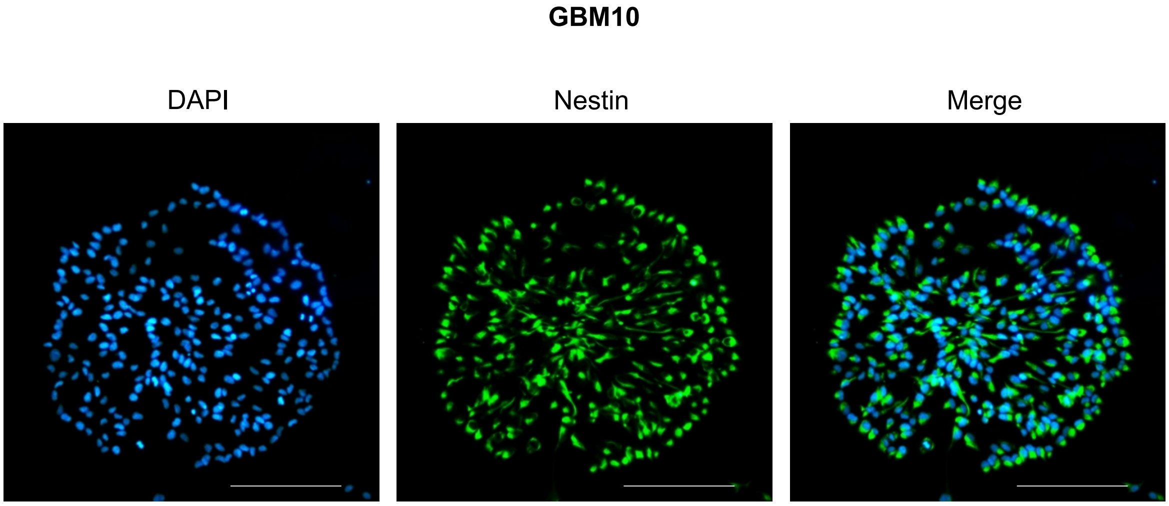

B

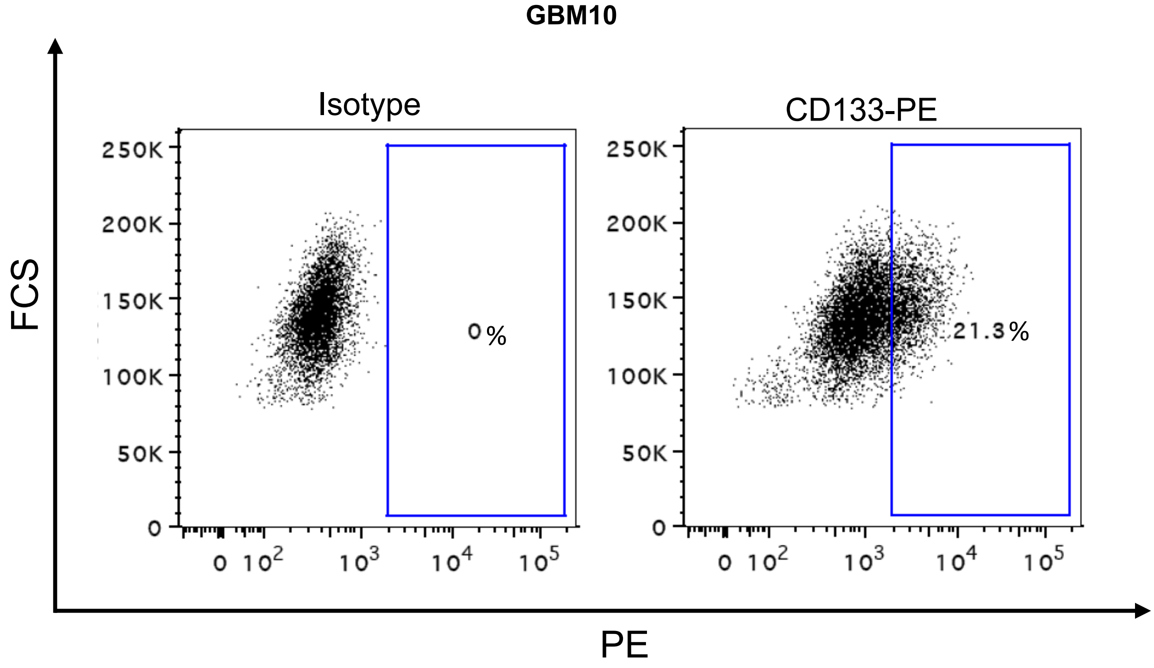

C

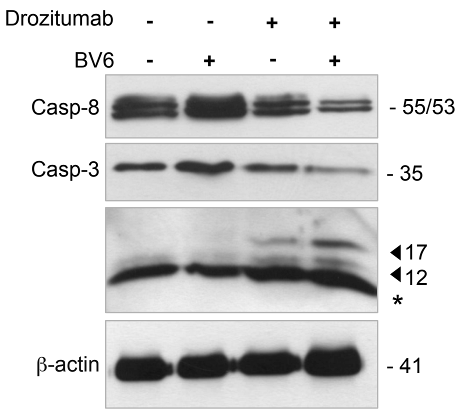

Suppl.Fig. 3

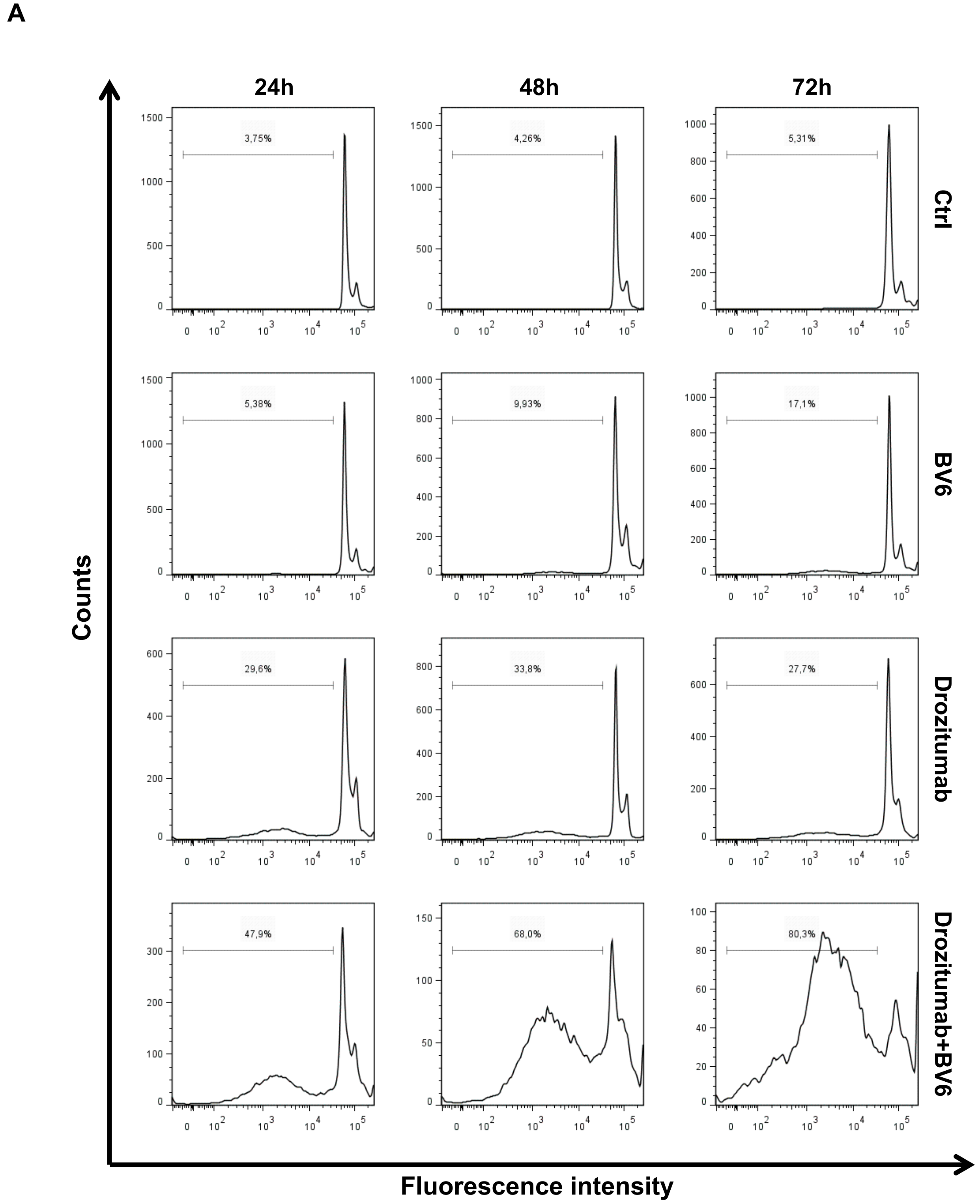
A
24h
48h
72h
Ctrl
BV6
Drozitumab
Drozitumab+BV6
3,75%
4,26%
5,31%
5,38%
9,93%
17,1%
29,6%
33,8%
27,7%
47,9%
68,0%
80,3%
Counts
Fluorescence intensity

Suppl.Fig. 3

B

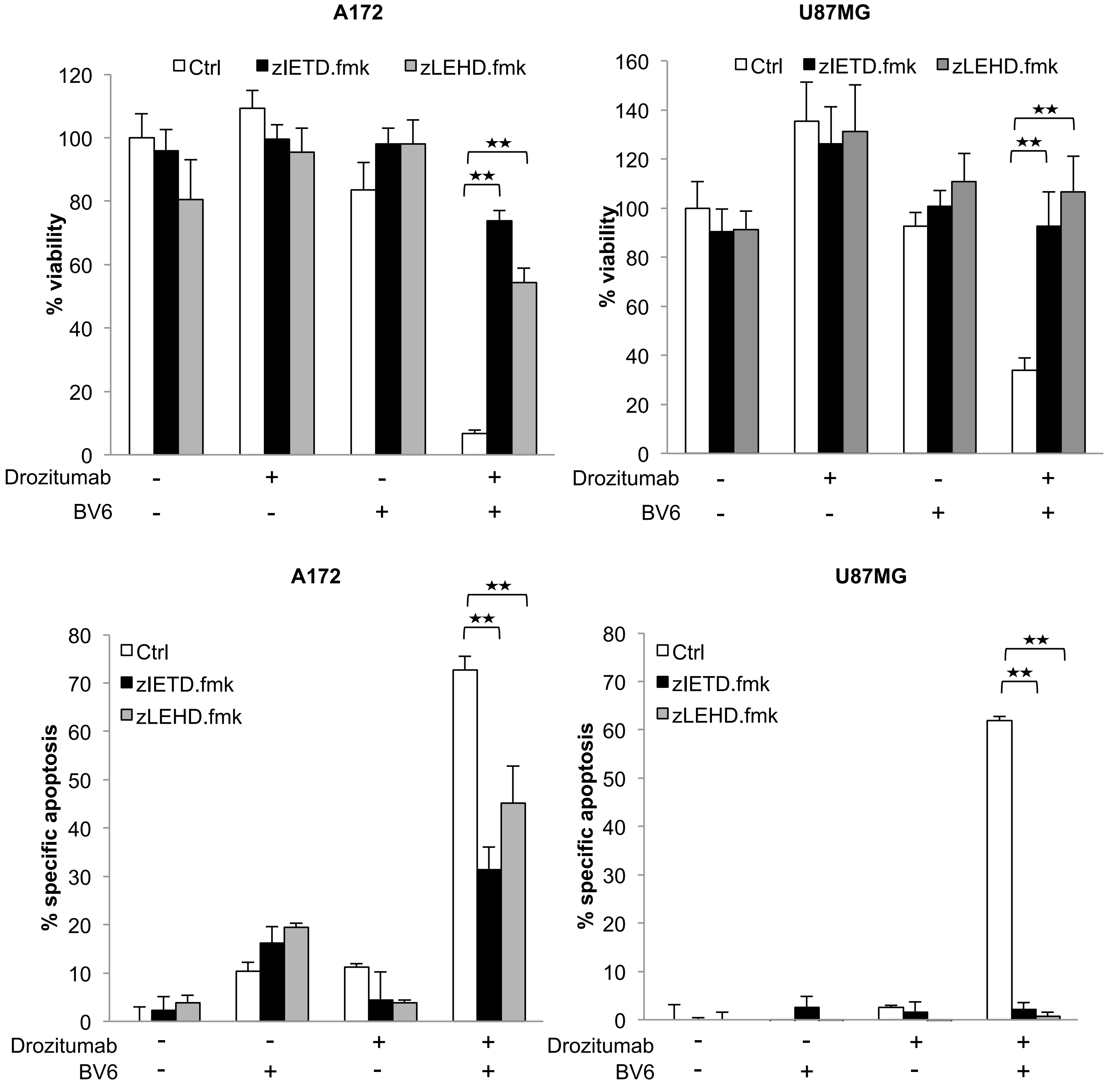

A172
Ctrl
zIETD.fmk
zLEHD.fmk
% viability
120
100
80
60
40
20
0
Drozitumab - + - +
BV6 - - + +
U87MG
160
140
120
100
80
60
40
20
0
A172
% specific apoptosis
80
70
60
50
40
30
20
10
0
Drozitumab - - + +
BV6 - + - +
U87MG

Suppl.Fig. 3

C

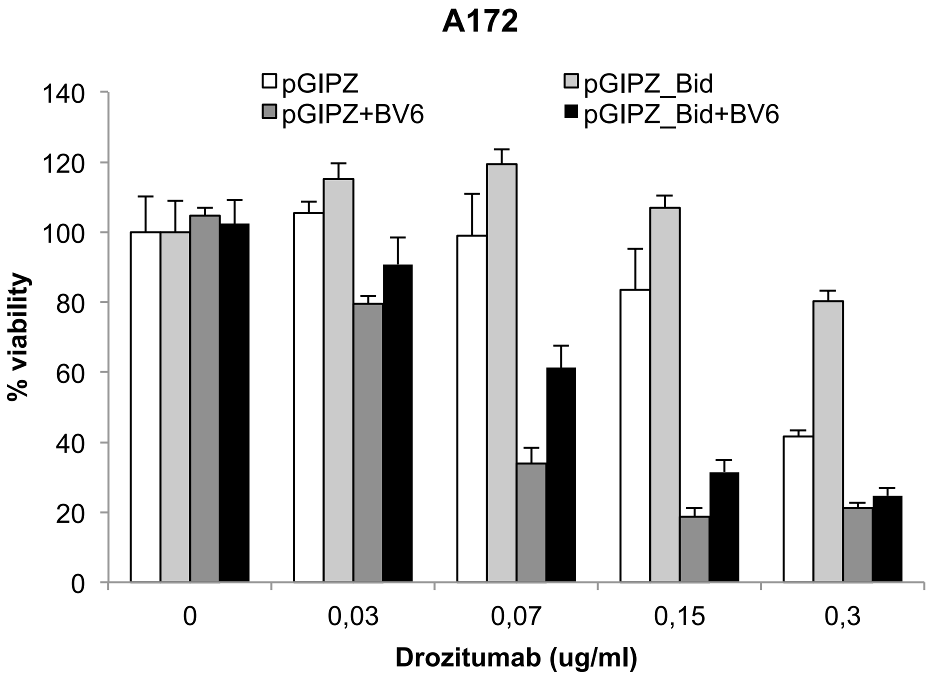

A172
pGIPZ
pGIPZ_Bid
pGIPZ+BV6
pGIPZ_Bid+BV6
140
120
100
80
60
40
20
0
% viability
0
0,03
0,07
0,15
0,3
Drozitumab (ug/ml)

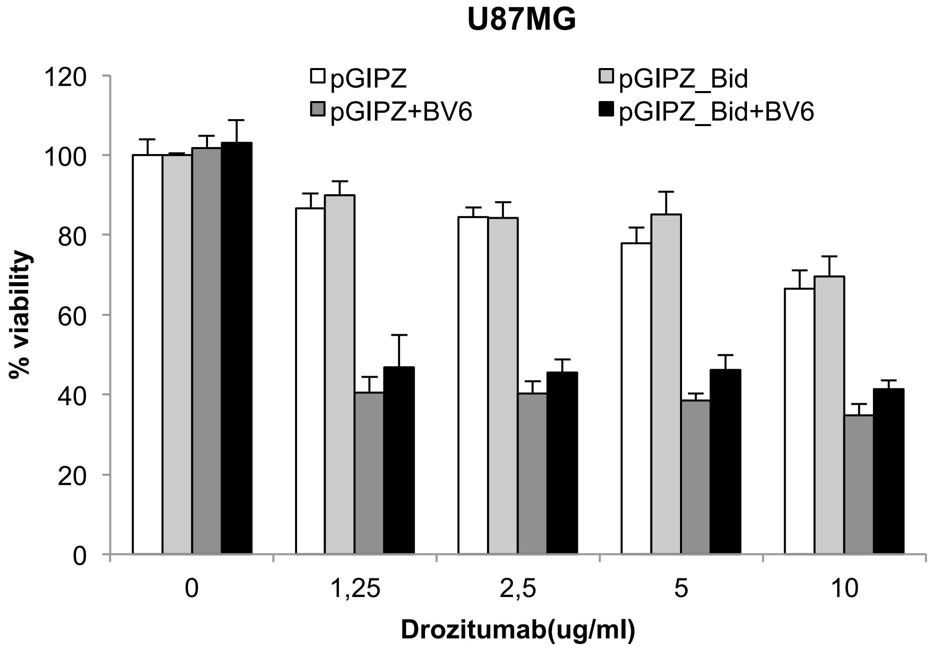

U87MG
pGIPZ
pGIPZ_Bid
pGIPZ+BV6
pGIPZ_Bid+BV6
120
100
80
60
40
20
0
% viability
0
1,25
2,5
5
10
Drozitumab(ug/ml)

Suppl.Fig. 4

A

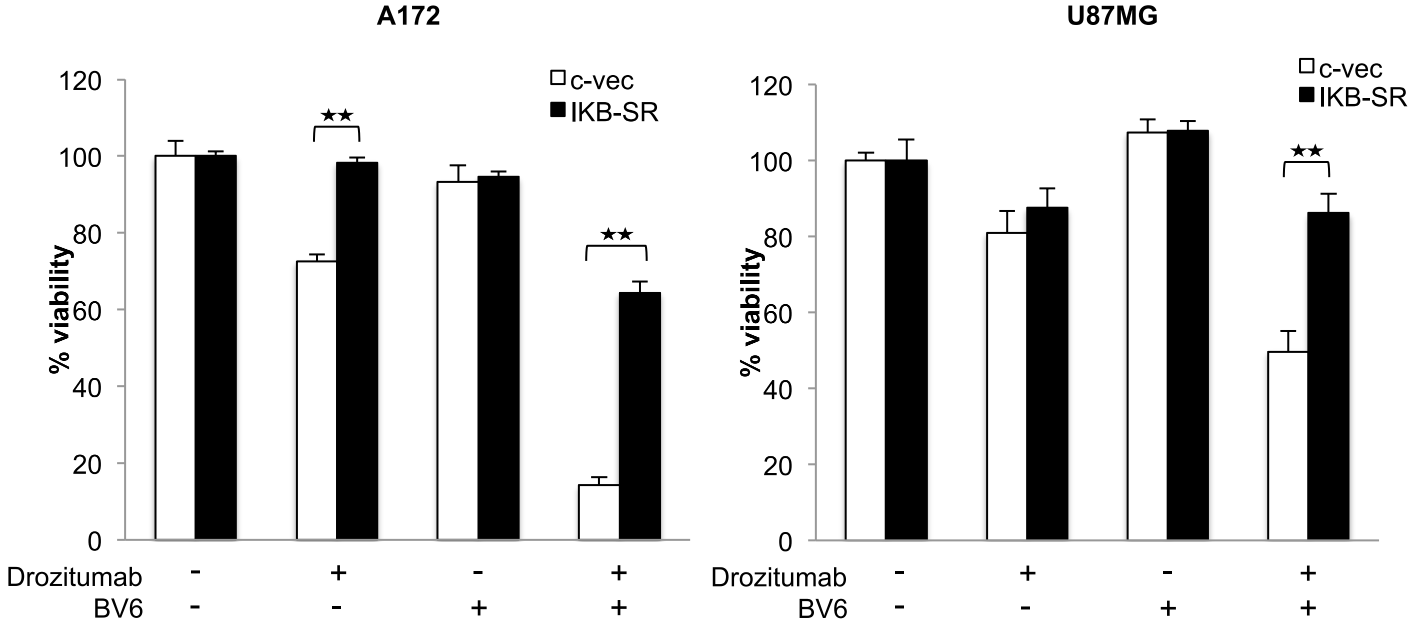

B

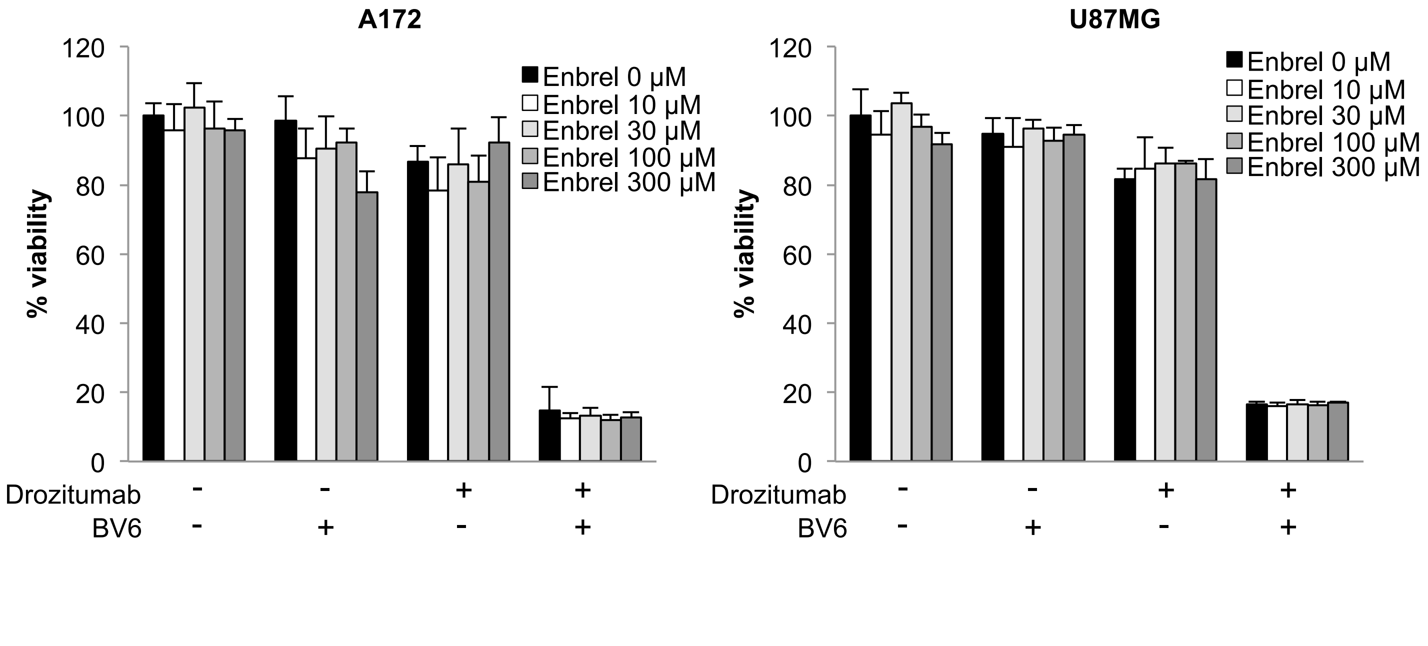

Suppl.Fig. 5

A

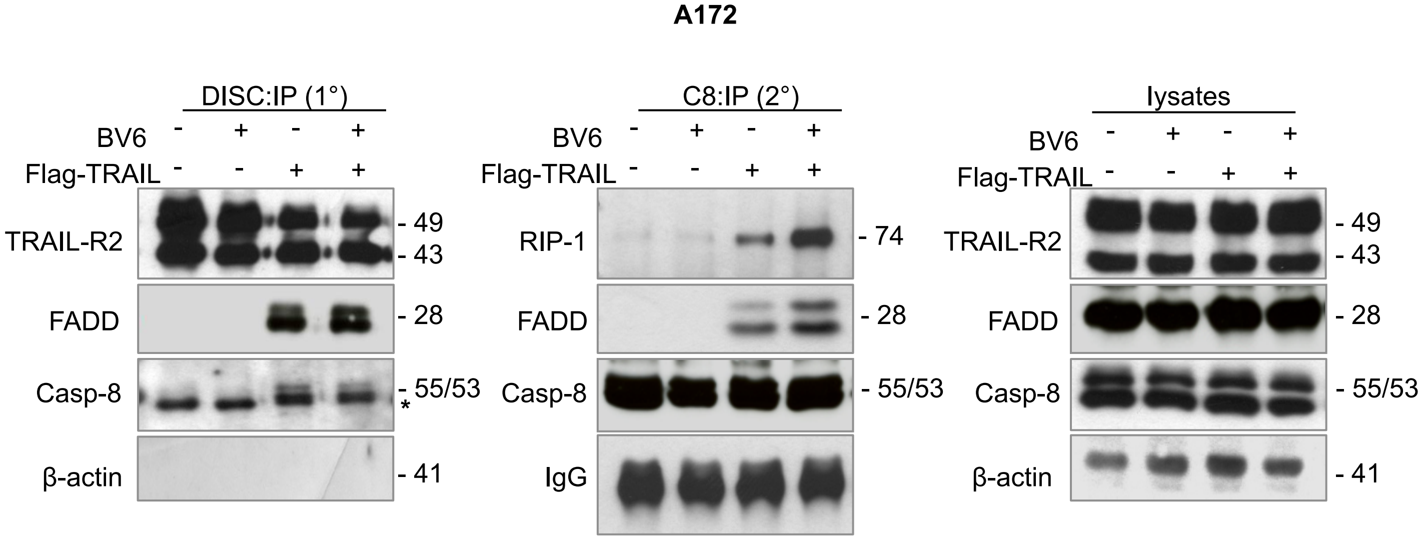

B

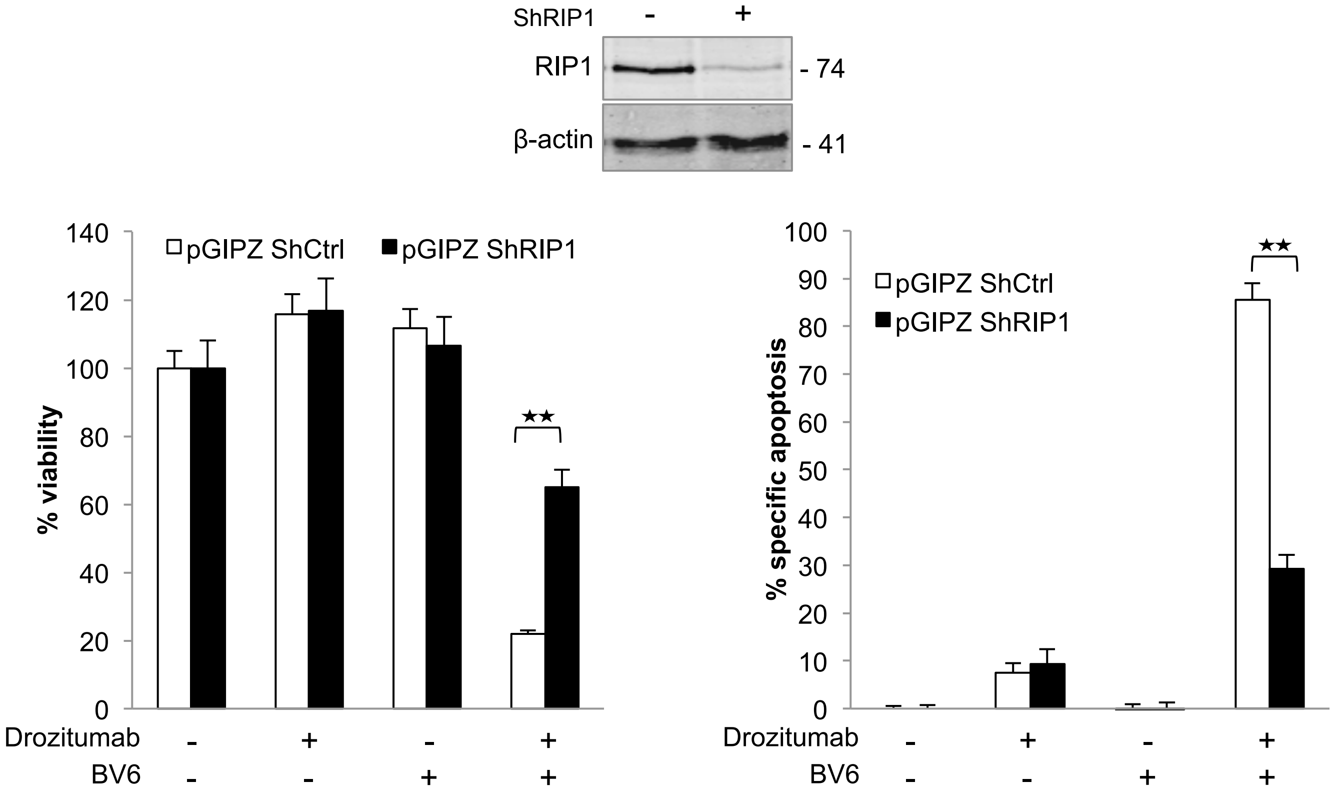

Suppl.Fig. 5

C

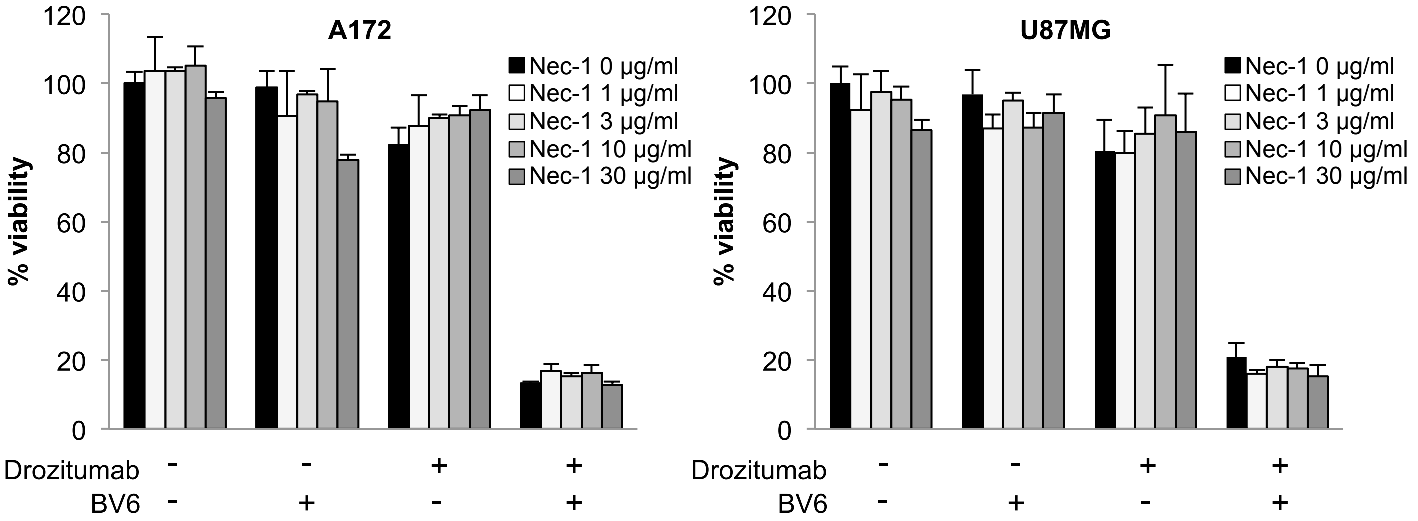

D

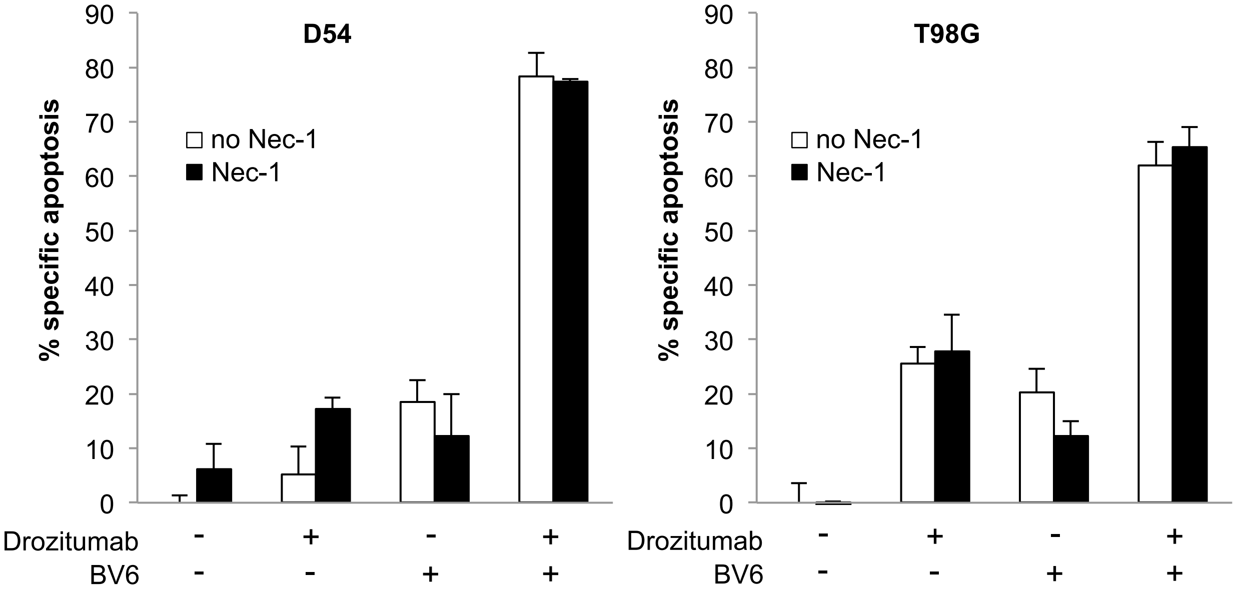

E

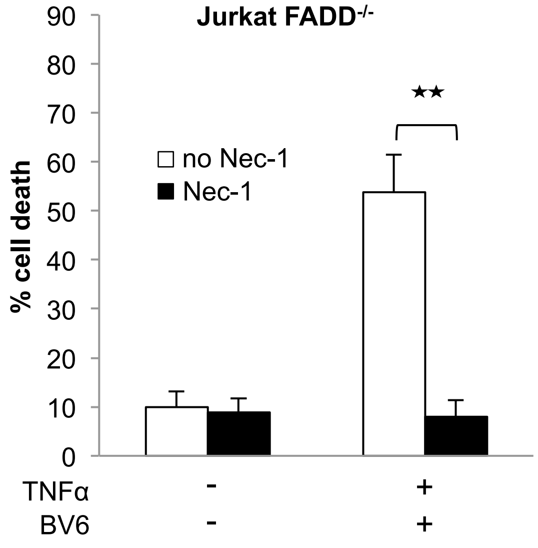

F

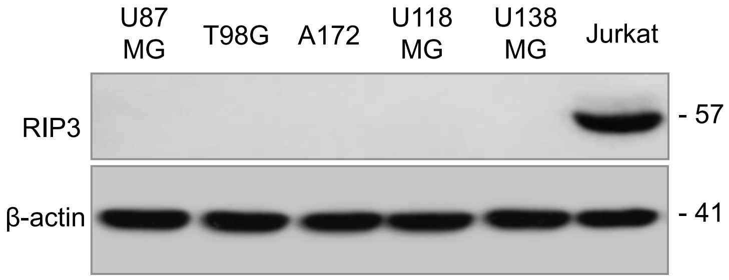

Supplementary Figure legends

Suppl. Tab. 1. p53 and PTEN status of GBM cell lines used in the study.

p53 and PTEN status (wild type, mutated) is shown as previously reported by [1].

Suppl. Fig. 1 Smac mimetic sensitizes glioblastoma cells to Drozitumab-induced apoptosis.

A, Surface expression of TRAIL-R1 and -R2 on T98G cells was determined by flow cytometry, isotype antibody was used to control unspecific antibody binding. A representative histograms of three independent experiments is shown, fluorescence intensity is plotted against cell counts.

B, D54 (left panel) and LN229 (right panel) cells were treated for 72 hours with indicated concentrations of Drozitumab and/or BV6. Cell viability was determined by MTT assay and is expressed as percentage of untreated controls. Data represent mean + SEM of three independent experiments performed in triplicate; ** $P < 0.001$ comparing samples treated with the combination versus samples treated with Drozitumab alone).

C, MiaPaCa pancreatic carcinoma cells were treated for 48 hours with 0.1 µg/ml Drozitumab and/or 2 µM BV6. Cell viability was determined by MTT assay and is expressed as percentage of untreated controls. Data represent mean + SEM of three independent experiments performed in triplicate.

D, U87MG cells were seeded on the CAM of chicken embryos and treated with 1 µM BV6 and/or 2.5 µg/ml Drozitumab for two days. Tumor growth was analyzed using hematoxylin and eosin-stained paraffin sections of the CAM as described in Materials and Methods. Representative pictures of hematoxylin and eosin-stained sections of the CAM are shown at 40x magnification.

Suppl. Fig. 2 Smac mimetic sensitizes glioblastoma stem cells to Drozitumab-induced apoptosis.

A, Nestin expression (green) was detected by immunofluorescence microscopy in GBM10 spheres. Nuclei were counterstained with DAPI (blue); scale bar: 500 µm.

B, CD133 expression of GBM10 cells was determined by FACS analysis.

C, Primary glioblastoma stem-like cells (GBM10) were treated for 48 hours with 10 µg/ml Drozitumab and/or 2.5 µM BV6. Caspase activation was analyzed by Western blotting; arrowheads indicate cleavage fragments, the asterisk indicates an unspecific band. A representative experiment of two independent experiments is shown.

Suppl. Fig. 3 BV6 cooperates with Drozitumab to trigger caspase-dependent apoptosis.

A, A172 cells were treated for 24, 48 and 72 hours with 0.3 µg/ml Drozitumab and/or 3 µM BV6. DNA fragmentation of propidium iodide-stained nuclei was determined by FACS analysis. Representative histograms of three independent experiments are shown for determination of DNA fragmentation of propidium iodide-stained nuclei by FACS analysis.

B, A172 and U87MG cells were treated for 72 hours with Drozitumab (U87MG: 5 µg/ml; A172, 0.3 µg/ml) and/or BV6 (U87MG: 4 µM; A172: 3 µM) in the presence or absence of 20 µM zIETD.fmk or zLEHD.fmk. Cell viability was determined by MTT assay and is shown as the percentage of untreated controls (upper panels). Apoptosis was determined by FACS analysis of DNA fragmentation of propidium iodide-stained nuclei and is shown as specific apoptosis (lower panels). Data represent mean + SEM of three independent experiments performed in triplicate; **, $P < 0.001$.

C, U87MG and A172 cells with knockdown of BID were treated for 72 hours with indicated concentrations of Drozitumab and/or BV6 (U87MG: 4 µM; A172: 3 µM). Cell viability was determined by MTT assay and is shown as the percentage of untreated controls. Data represent mean + SEM of three independent experiments performed in triplicate.

Suppl. Fig. 4 TNFα is dispensable for Smac mimetic- and Drozitumab-induced apoptosis.

A, Glioblastoma cell lines overexpressing IκBα-SR were treated 72 hours with Drozitumab (U87MG: 10 µg/ml; A172: 0.6 µg/ml) and/or BV6 (U87MG: 4 µM; A172: 3 µM). Cell viability was determined by MTT assay and is expressed as percentage of untreated controls. Data represent mean + SEM of three independent experiments performed in triplicate; ** $P < 0.001$.

B, Glioblastoma cell lines were pretreated or not for 1 hour with 100 µg/ml Enbrel and then treated for 72 hours in the presence or absence of indicated concentrations of Enbrel with 0.3 ng/ml (A172) or 5 ng/ml (U87MG) Drozitumab and/or BV6 (U87MG: 4 µM; A172: 3 µM). Cell viability was determined by MTT assay and is expressed as percentage of untreated controls. Data represent mean + SEM of three independent experiments performed in triplicate.

Suppl. Fig. 5 Effect of BV6 on Drozitumab-induced TRAIL DISC formation and role of RIP1.

A, A172 cells were pretreated or not with 3 µM BV6 for 30 minutes and then treated with Flag-tagged TRAIL for 1 hour. TRAIL DISC was analyzed by immunoprecipitation and Western blotting of caspase-8, FADD and TRAIL-R2 as described in Materials and Methods. In the DISC-depleted fraction, caspase-8 was

subsequently immunoprecipitated (C8:IP) using an anti-caspase-8 antibody and indicated proteins were detected by Western blot analysis.

B, U87MG cells were transduced with pGIPZ shRNA vector against RIP1 (ShRIP1) or control vector (ShCtrl). Expression of RIP1 was analyzed by Western blotting (upper part). Cells were treated for 72 hours with 5 ng/ml Drozitumab and/or 4 μM BV6. Cell viability was determined by MTT assay and is shown as the percentage of untreated controls (B, left lower panel). Apoptosis was determined by FACS analysis of DNA fragmentation of propidium iodide-stained nuclei and is shown as specific apoptosis (B, right lower panel). Data represent mean + SEM of three independent experiments performed in triplicate; **, $P < 0.001$.

C, A172 and U87MG cells were treated for 72 hours in the presence or absence of indicated concentrations of Necrostatin-1 with 0.3 ng/ml (A172) or 5 ng/ml (U87MG) Drozitumab and/or 3 μM (A172) or 4 μM (U87MG) BV6. Cell viability was determined by MTT assay and is expressed as percentage of untreated controls. Data represent mean + SEM of three independent experiments performed in triplicate; **, $P < 0.001$.

D, D54 and T98G cells were treated for 72 hours with 0.3 ng/ml Drozitumab and/or 3 μM BV6 in the presence or absence of 30 μM Necrostatin-1. Apoptosis was determined by FACS analysis of DNA fragmentation of propidium iodide-stained nuclei and is shown as specific apoptosis. Data represent mean + SEM of three independent experiments performed in triplicate; **, $P < 0.001$.

E, Jurkat FADD-/- cells were treated for 4 hours with 1 ng/ml TNFα and 1 μM BV6 in the presence or absence of 30 μM Necrostatin-1. Cell death was determined by FACS analysis of propidium iodide-positive cells. Data represent mean + SEM of three independent experiments performed in triplicate; **, $P < 0.001$.

F, Protein expression of RIP3 was assessed by Western blot analysis in several glioblastoma cell lines, Jurkat cells were used as positive control for RIP3 expression. β-actin was used as loading control.

Supplementary Materials and Methods

Cells

A172 and U87MG cells with stable overexpression of IκBα-SR or knockdown of Bid have previously been described [2, 3].

Nestin and CD133 staining.

GBM10 spheres were put on poly-ornithine-coated plates for 24h, fixed with 4% paraformaldehyde for 10 min, permeabilized with 0.1% Triton X-100 for 10 min and stained at room temperature with human anti-Nestin antibody (Abcam, Cambridge, UK) followed by FITC-labeled goat anti-mouse IgG (H+L) (Jackson ImmunoResearch, Newmarket, Suffolk, UK) supplemented with DAPI to counterstain nuclei. Pictures were taken by Olympus IX71 microscope (Olympus GmbH, Hamburg, Germany). CD133 expression was analyzed by FACS analysis using CD133/2 antibody (Miltenyi Biotech, Bergisch Gladbach, Germany) according to the manufacturer's instructions.

Immunoprecipitation and Western blotting.

Immunoprecipitation of the TRAIL DISC was performed as previously described [4]. TRAIL DISC immunoprecipitation was followed by caspse-8 immunoprecipitation. To this end, 1 mg of protein from the DISC-depleted fraction was incubated with 10 µg mouse anti-caspase-8 antibody (Alexis, Grünberg, Germany) overnight at 4° C followed by the addition of 20 µl pan-mouse IgG Dynabeads (Invitrogen), then incubated for two hours at 4° C and washed with NP40 buffer. Protein expression of caspase-8, RIP1 and FADD was detected by Western blotting.

RNA interference.

HEK293T producer cells were transfected with 7.5 µg pGIPZ-shRNAmir vector, 12.5

µg pCMV-dR8.91 and 1 µg pMD2.G using calcium phosphate transfection. All pGIPZ-shRNAmir-vectors were purchased from Thermo Fisher Scientific (Waltham, MA): non-silencing control (ShCtrl): RHS4346, ShRIP1: RHS4430-101068916. Virus-containing supernatant was collected after 48 hours and filtered using a 45 µm filter. U87MG cells were transduced by centrifugation at 1000xg for 1 hour at room temperature in the presence of 8 µg/ml polybrene and selected for two weeks with 1 µg/ml puromycin.

References:

1. Ishii N, Maier D, Merlo A, Tada M, Sawamura Y, Diserens AC*, et al.* Frequent co-alterations of TP53, p16/CDKN2A, p14ARF, PTEN tumor suppressor genes in human glioma cell lines. *Brain Pathol*. 1999; **9:** 469-479.

2. Jennewein C, Karl S, Baumann B, Micheau O, Debatin KM, Fulda S. Identification of a novel pro-apoptotic role of NF-kappaB in the regulation of TRAIL- and CD95-mediated apoptosis of glioblastoma cells. *Oncogene*. 2012; **31:** 1468-1474.

3. Cristofanon S, Fulda S. ABT-737 promotes tBid mitochondrial accumulation to enhance TRAIL-induced apoptosis in glioblastoma cells. *Cell Death Dis*. 2012; **3:** e432.

4. Mader I, Wabitsch M, Debatin KM, Fischer-Posovszky P, Fulda S. Identification of a novel proapoptotic function of resveratrol in fat cells: SIRT1-independent sensitization to TRAIL-induced apoptosis. *FASEB J*. 2010; **24:** 1997-2009.
